# MoS_2_–Plasmonic Hybrid Platforms: Next-Generation Tools for Biological Applications

**DOI:** 10.3390/nano15020111

**Published:** 2025-01-13

**Authors:** Nayra A. M. Moussa, Seungah Lee, Seong Ho Kang

**Affiliations:** 1 Basic and Clinical Medical Science Department, Faculty of Dentistry, Deraya University, New Minya 61768, Egypt; nayramoussa8@gmail.com; 2 Department of Applied Chemistry and Institute of Natural Sciences, Kyung Hee University, Yongin-si 17104, Gyeonggi-do, Republic of Korea

**Keywords:** MoS_2_–plasmonic hybrids, biosensing, bioimaging, phototherapy

## Abstract

The combination of molybdenum disulfide (MoS_2_) with plasmonic nanomaterials has opened up new possibilities in biological applications by combining MoS_2_’s biocompatibility and high surface area with the optical sensitivity of plasmonic metals. These MoS_2_–plasmonic hybrid systems hold great promise in areas such as biosensing, bioimaging, and phototherapy, where their complementary properties facilitate improved detection, real-time visualization, and targeted therapeutic interventions. MoS_2_’s adjustable optical features, combined with the plasmon resonance of noble metals such as gold and silver, enhance signal amplification, enabling detailed imaging and selective photothermal or photodynamic therapies while minimizing effects on healthy tissue. This review explores various synthesis strategies for MoS_2_–plasmonic hybrids, including seed-mediated growth, in situ deposition, and heterojunction formation, which enable tailored configurations optimized for specific biological applications. The primary focus areas include highly sensitive biosensors for detecting cancer and infectious disease biomarkers, high-resolution imaging of cellular dynamics, and the development of phototherapy methods that allow for accurate tumor ablation through light-induced thermal and reactive oxygen species generation. Despite the promising advancements of MoS_2_–plasmonic hybrids, translating these platforms into clinical practice requires overcoming considerable challenges, such as synthesis reproducibility, toxicity, stability in physiological conditions, targeted delivery, and scalable manufacturing. Addressing these challenges is essential for realizing their potential as next-generation tools in diagnostics and targeted therapies.

## 1. Introduction

The extensive use of nanoscale materials in biomedical science, biosensors, and biomolecular imaging for disease diagnosis, prevention, and treatment has attracted considerable interest from researchers [1,2,3,4,5,6,7]. The distinct properties of these materials—such as low toxicity, accurate targeting capabilities, and highly tunable features—make them well suited for various biomedical applications [8,9].

Nanomaterials can be categorized by dimensionality into zero-dimensional (0D), one-dimensional (1D), two-dimensional (2D), and three-dimensional (3D) structures [10]. Among these, 2D materials have attracted particular attention owing to their remarkable physicochemical properties and extensive potential applications. Their atomically thin layers provide thermal stability, mechanical strength, and flexibility [11,12,13], which are advantageous for biomedical and sensing applications.

Since the isolation of graphene in 2004 by Novoselov et al. [14], a diverse array of 2D nanomaterials, including black phosphorus, boron nanosheets, antimonene, and hexagonal boron nitride, has been investigated [15,16,17,18,19,20,21,22]. Transition metal dichalcogenides (TMDs), particularly molybdenum disulfide (MoS_2_), have emerged as promising alternatives to graphene, presenting unique optical, electrical, and chemical properties that are advantageous for various biomedicine applications [23,24,25,26,27,28]. Nanostructured MoS_2_-based biomaterial features a distinctive direct bandgap, strong near-infrared (NIR) absorbance, large surface area, and readily functionalizable sites, showing considerable potential in fields such as biosensing [29,30,31,32], bioimaging [33], phototherapy [34,35,36], drug delivery [37,38], and theranostics [39,40].

Moreover, the minimal cytotoxicity and excellent biocompatibility of MoS_2_ provide additional advantages over graphene and other materials. Teo et al. conducted cell viability assessments on human lung carcinoma epithelial cells (A549) exposed to various concentrations of TMDs and graphene over 24 h [41]. The results showed that MoS_2_ nanosheets maintained cell viability levels at least twice as high as those of graphene, even at concentrations 1.6 times greater than those of graphene. Notably, MoS_2_ nanomaterials exhibited considerably fewer hazardous effects compared with graphene oxides and halogenated graphene, establishing MoS_2_ as a more biocompatible option for potential biomedical applications.

While graphene’s zero-bandgap limits its suitability for electronic and optoelectronic applications, MoS_2_ exhibits compelling properties, including a substantial direct bandgap of approximately 1.9 eV in its monolayer form. This bandgap is tunable and increases with decreasing number of layers, which is attributed to quantum confinement effects. Bulk MoS_2_ has an indirect bandgap of approximately 1.2 eV, which transitions to a direct bandgap of approximately 1.9 eV when reduced to a monolayer. These unique characteristics make MoS_2_ a highly attractive candidate, offering advantages over its counterparts for various biosensing applications. Similarly, black phosphorus-based photodetectors demonstrate exceptional photodetection capabilities owing to their suitable bandgap and high carrier mobility. However, their poor stability severely restricts their use in long-term photodetection applications [42].

Notably, the hybridization of MoS_2_ with plasmonic materials (e.g., gold and silver nanoparticles) is a groundbreaking approach that merges the complementary properties of both components. MoS_2_–plasmonic hybrids leverage the unique plasmonic features of metallic nanoparticles (NPs) to enhance electronic and optical signals, resulting in substantial improvements in sensitivity for biosensing and advanced imaging capabilities for super-resolution bioimaging. These hybrid platforms also facilitate localized surface plasmon resonance (LSPR)-based manipulation, creating new opportunities for real-time biological sensing and targeted therapeutics [43].

Regarding LSPR applications, a systematic study demonstrated that MoS_2_ offers greater favorability than graphene [44]. MoS_2_ exhibits robust light–matter interactions, which substantially enhance the sensitivity of biosensors when layered with gold nanoparticles (AuNPs). This configuration achieves sensitivity of up to 403 nm/RIU, highlighting MoS_2_’s superior performance in molecular detection. Although graphene exhibits excellent electrical conductivity and absorption properties, its relatively low absorption per atomic layer (~2.3%) limits its effectiveness in enhancing sensitivity for LSPR applications. In contrast, MoS_2_ exhibits stronger absorption, with an absorption per atomic layer of approximately 5%. Moreover, MoS_2_’s compatibility in hybrid structures establishes it as a more effective choice for advancing LSPR technology, particularly for ultrasensitive biosensing applications.

This synergy between MoS_2_ and plasmonic materials enhances their functional versatility, positioning them as ideal candidates for next-generation tools in biological applications, such as high-sensitivity biosensors, image-guided therapy, and diagnostic platforms. By combining MoS_2_’s inherent biocompatibility with the signal-enhancing properties of plasmonic NPs, these hybrid materials offer promising solutions to complex biomedical challenges that require accurate detection and real-time imaging capabilities [45].

Previous studies have advanced our understanding of MoS_2_ interactions with plasmonic nanostructures. While some reviews have focused on the synthesis and applications of noble metal nanostructure-decorated MoS_2_ hybrids, this review aims to elucidate the mechanisms underlying the interactions between MoS_2_ and plasmonic materials, highlighting their enhanced electronic, optical, and catalytic properties. In addition, this review addresses the challenges of harnessing the innovative potential of MoS_2_–plasmonic hybrids as next-generation platforms for various biological applications.

## 2. MoS_2_ Nanomaterials

### 2.1. MoS_2_ Structure

**Figure 1:**
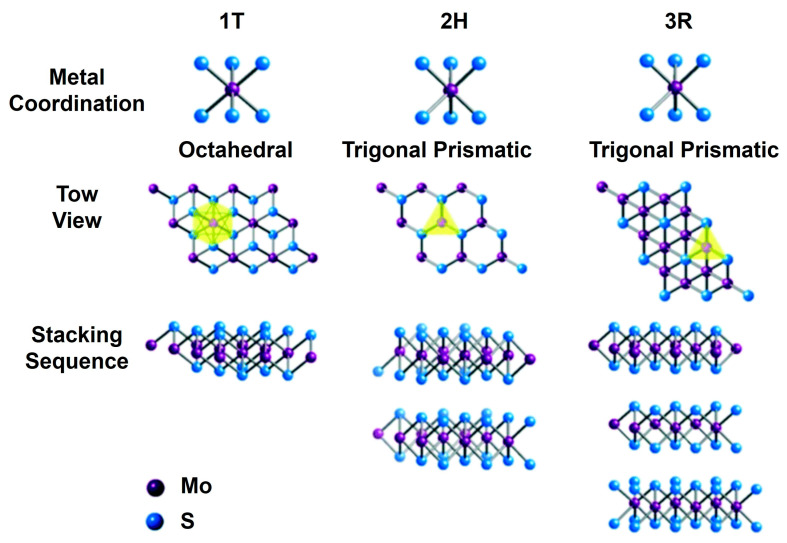
An illustration of the different metal coordination geometries and stacking sequences of the three distinct phases of MoS_2_. (**Top Row**) 1T Phase: Exhibits octahedral coordination of the Mo atoms, represented by purple spheres, with S atoms depicted as blue spheres; 2H Phase: Features trigonal prismatic coordination, where the Mo atoms are surrounded by six S atoms arranged in a prism-like structure; and 3R Phase: Displays trigonal prismatic coordination, similar to the 2H phase but with a different stacking arrangement. (**Middle Row**) Top view of each phase, showing the arrangement of the Mo and S atoms in the respective structures. Yellow triangles indicate the coordination environment around the Mo atoms. (**Bottom Row**) Stacking sequence of the Mo and S layers for each phase, showing arrangement of layers in three-dimensional space. Reprinted with permission from Ref. [46]. Copyright (2017) Royal Society of Chemistry.

Two-dimensional MoS_2_ nanomaterials exist in different layer configurations and structural forms. A single-layer MoS_2_ usually displays either an octahedral or a trigonal prismatic coordination phase, as shown in Figure 1 [46]. In multilayered MoS_2_ nanostructures, each layer can independently assume either phase, resulting in a variety of polymorphic structures. The most prevalent polymorphs are trigonal (1T), hexagonal (2H), and rhombohedral (3R), with the numeral indicating the coordination number [46,47].

The 1T phase of MoS_2_ displays metallic characteristics, while the 2H and 3R phases possess semiconducting properties, making them suitable for a range of electronic applications. Furthermore, both the 2H and 3R phases serve effectively as dry lubricants, and the nonlinear optical properties of the 3R phase enhance its applicability in nonlinear optical mass sensing, quantum measurements, and biomedicine [48]. Among the different stacking configurations, the 1H MoS_2_ phase is the most stable, featuring a sandwich-like S–Mo–S structure, where each sulfur (S) layer is covalently bonded to a Mo layer, resulting in a thickness of approximately 0.65 nm [49]. The adjacent S–Mo–S layers are held together by weaker van der Waals forces [50]. The conductivity of MoS_2_ is influenced by both temperature and thickness, generally increasing with temperature but decreasing as thickness increases, ultimately approaching the properties of bulk material [51].

MoS_2_ exhibits remarkable versatility, appearing in two-dimensional structures (e.g., nanosheets, nanoribbons), one-dimensional configurations (e.g., nanowires, nanotubes), and zero-dimensional forms (e.g., quantum dots, nanoplatelets). For instance, 2D nanoribbons generally measure one to three layers in thickness, while one-dimensional nanowires (NWs) are approximately 14–30 nm in length and 0.6 nm in width [52]. Previously studied one-dimensional nanoplatelets range in size from 12 to 30 nm, with a width roughly equivalent to one unit cell, and demonstrate exceptional catalytic activity in hydrodesulfurization [53]. In contrast, quantum dots vary from 2 to 10 nm, possessing a larger bandgap than nanosheets and showcasing stronger Mo–Mo bonding compared to monolayers.

The transition of MoS_2_ nanomaterials from two-dimensional to lower-dimensional structures results in changes to the bandgap, affecting both photoluminescence (PL) characteristics and optical properties. Monolayers and other low-dimensional forms of MoS_2_ can seamlessly integrate into optical nanostructures, where light–matter interactions enhance PL intensity and emission rates [54,55,56]. These improvements underscore the potential of MoS_2_ in advanced optical applications [33].

### 2.2. MoS_2_ Properties

#### 2.2.1. Electronic Properties

**Figure 2:**
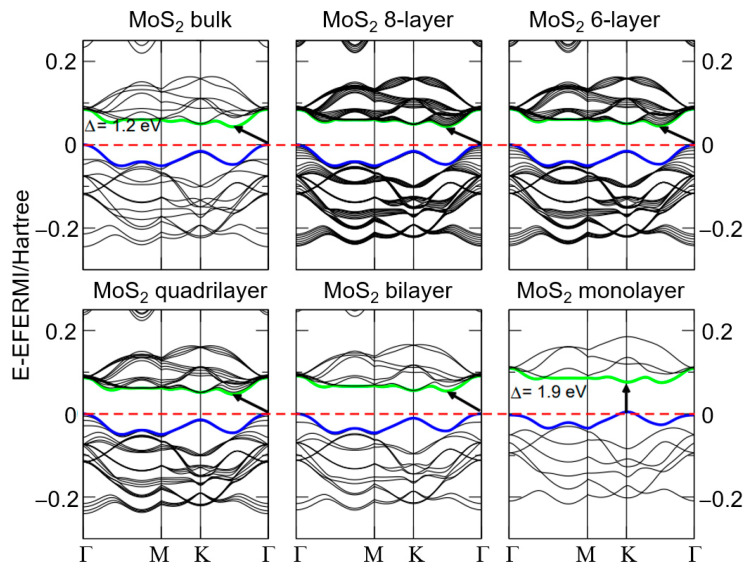
Band structure plots of the electron levels of bulk, multilayer, and monolayer MoS_2_. Green and blue lines denote the conduction and valence bands, respectively, while the red dashed lines indicate the Fermi level. The small bold arrows represent the bandgap values for each system. Reprinted with permission from Ref. [58]. Copyright (2011) American Physical Society.

MoS_2_ nanomaterials display unique electronic properties. In its bulk and multilayer forms, MoS_2_ has an indirect bandgap, which increases as the number of layers decreases, ultimately reaching a direct bandgap of 1.8 eV in monolayer MoS_2_ [57]. This inverse relationship between the bandgap and the number of MoS_2_ layers is clearly shown in Figure 2 [58]. As shown in the first row of Figure 2, the bulk MoS_2_ structure exhibits an indirect bandgap of approximately 1.2 eV, while the eight-layer configuration also maintains an indirect bandgap, following a similar energy trend. The six-layer MoS_2_ structure still maintains an indirect bandgap. However, energy dispersion indicates modifications in electronic properties as the number of layers decreases. In the second row of Figure 2, the quadrilateral MoS_2_ band structure transitions toward a direct bandgap with a clear separation between the valence and conduction bands. The MoS_2_ bilayer further confirms this transition to a direct bandgap, with the gap increasing as the number of layers decreases. Finally, the MoS_2_ monolayer reveals a direct bandgap of approximately 1.9 eV, highlighting the notable changes in electronic properties as the material transitions from bulk to monolayer. Comparatively, this direct bandgap remains higher than the 1.12 eV indirect bandgap of silicon [59], making MoS_2_ a promising candidate for electronic applications.

#### 2.2.2. Optical Properties

The optical properties of MoS_2_, especially its absorption coefficient and refractive index (RI), are vital for optoelectronic applications. The absorption coefficient indicates the depth of light penetration before absorption and is high for MoS_2_ in the 400–500 nm wavelength range, with a considerable decline occurring around 500 nm [60]. MoS_2_’s adjustable bandgap, influenced by its size and structure, allows for tunable photoresponsivity, detectivity, and response time [61]. Both monolayer and multilayer MoS_2_ structures demonstrate a high RI (>2), making them suitable for coatings.

**Figure 3:**
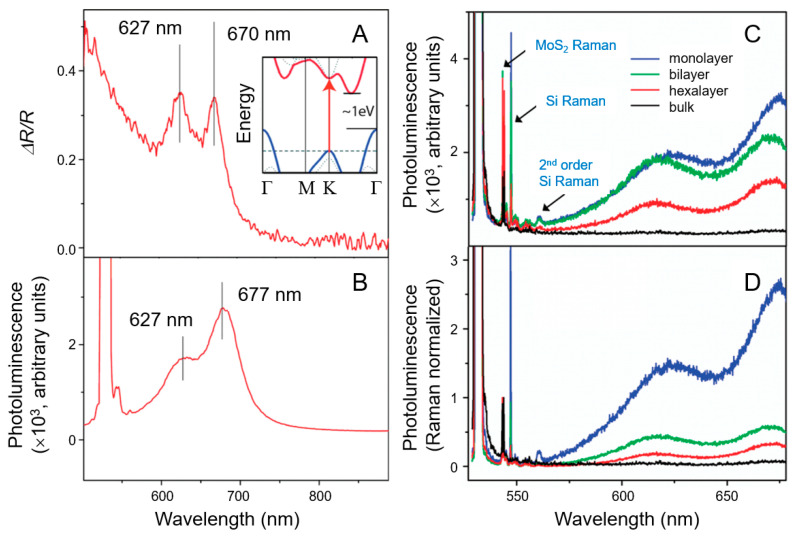
(**A**) The reflection difference observed from an ultrathin MoS_2_ layer on a quartz substrate is proportional to the MoS_2_ absorption coefficient. The absorption peaks at 1.85 eV (670 nm) and 1.98 eV (627 nm) can be attributed to the direct excitonic transitions of A_1_ and B_1_, resulting from energy splitting owing to valence band spin–orbit coupling. The inset shows the bulk MoS_2_ band structure, excluding the comparatively weak spin–orbit coupling, which exhibits an indirect bandgap of approximately 1 eV and a single higher-energy direct excitonic transition near the K point, indicated by an arrow. (**B**) A pronounced photoluminescence (PL) peak is observed at the energies of direct excitonic transitions in monolayer MoS_2_, whereas the indirect bandgap bulk MoS_2_ sample lacks such luminescence. (**C**) PL and Raman spectra of monolayer, bilayer, hexalayer, and bulk MoS_2_. Various Raman peaks correspond to the vibrational modes of MoS_2_ and silicon. The Raman signal from the MoS_2_ monolayer is faint owing to the limited material being stimulated. However, the PL intensity is most pronounced in monolayer MoS_2_ despite the reduced material. (**D**) PL spectra normalized to Raman intensity for MoS_2_ layers of varying thicknesses, demonstrating a substantial increase in luminescence efficiency for the MoS_2_ monolayer. Reprinted with permission from Ref. [65]. Copyright (2010) American Chemical Society.

Layer-dependent variations in the PL spectra of MoS_2_ offer further insights into its optical characteristics. Due to its direct bandgap, MoS_2_ efficiently absorbs photons with energies exceeding the bandgap. This results in a noticeable change in the PL spectra of ultrathin film MoS_2_, particularly evident in monolayer MoS_2_, as shown in Figure 3 [62,63,64,65]. This characteristic makes MoS_2_ a promising candidate for optoelectronic applications [66].

Figure 3A–C provide detailed insights into the reflection and PL spectra of ultrathin MoS_2_ and the layer-dependent effects on its PL efficiency. Notably, the ultrathin MoS_2_ layer on a quartz substrate exhibited prominent peaks in both the reflection and PL spectra, corresponding to the MoS_2_ absorption constant (Figure 3A). The absorption peaks at 1.85 eV (670 nm) and 1.98 eV (627 nm) are linked to the A_1_ and B_1_ direct excitonic transitions, resulting from the energy difference caused by spin–orbit coupling in the valence band. In the inset of Figure 3A, the band structure of bulk MoS_2_, which omits the relatively weak effect of spin–orbit coupling, reveals an indirect bandgap around 1 eV and a distinct, higher-energy direct excitonic transition near the K point, indicated by an arrow. Figure 3B shows a pronounced PL response at the energies of direct excitonic transitions in monolayer MoS_2_, which is lacking in the bulk MoS_2_ sample because of its indirect bandgap. To further investigate the optical properties of MoS_2_ nanomaterials, the layer-dependent PL efficiency of MoS_2_ has been extensively studied, with spectra obtained for monolayer, bilayer, hexalayer, and bulk samples, as illustrated in Figure 3C,D [65]. Clear Raman peaks were detected for both MoS_2_ and silicon vibrational modes. The Raman signal from thin-layer MoS_2_, especially the monolayer, is relatively weak owing to the limited amount of excited material. However, despite this reduced material representation, monolayer MoS_2_ demonstrates the highest PL intensity. An analysis of the PL spectra normalized by Raman intensity across different MoS_2_ layer configurations reveals a substantial increase in luminescence efficiency in monolayer MoS_2_.

#### 2.2.3. Catalytic Properties

The catalytic behavior of MoS_2_ is attributed to its layered structure, which offers accessible active sites such as edge sites, sulfur vacancies, and grain boundaries. Edge sites are particularly active for hydrogen evolution reaction (HER), providing favorable conditions for hydrogen adsorption and desorption [67]. Sulfur vacancies further enhance catalytic activity by increasing the number of adsorption sites, with optimal performance observed at a vacancy density of 7–10% [68,69]. The intrinsic turnover frequencies (TOFs) for different active sites show that edge sites are the most catalytically efficient, while sulfur vacancies offer moderate activity, and grain boundaries contribute minimally to HER activity, highlighting the critical role of edge sites in MoS_2_’s catalytic performance.

Vijayan and Sandhyarani developed an innovative thermal processing method to synthesize a new heterostructure catalyst for HER based on MoS_2_, specifically molybdenum disulfide–molybdenum trioxide–rhenium oxide (MoS_2_–MoO_3_–Re_2_O_7_) [70]. This composite is derived from bulk MoS_2_, which is typically considered a weak catalyst for HER. However, the MoS_2_–MoO_3_–Re_2_O_7_ composite shows considerably enhanced catalytic performance compared to bulk MoS_2_, as demonstrated by both electrochemical and photoelectrochemical studies. Mott–Schottky analysis indicates the formation of a p–n heterojunction, which improves interfacial contact and aids in the separation and movement of photogenerated charge carriers, thus increasing photoelectrocatalytic efficiency. The catalyst’s exceptional stability is further confirmed through extensive cycling tests.

Xu et al. performed a comprehensive analysis of MoS_2_-based electrocatalysts for HER [71]. They found that the crystal structure, microstructure, surface, and interface of MoS_2_ were critical factors in enhancing catalytic performance by modifying electrical conductivity, surface wettability, active site density, and changes in Gibbs free energy changes for hydrogen adsorption (ΔG_H_). Additionally, surface and interface engineering—encompassing phase engineering, defect engineering, morphology design, and heterostructure development—are identified as advanced strategies for creating MoS_2_-based catalysts that offer excellent efficacy at a low cost by optimizing surface-active sites, charge transfer, DGH, and surface hydrophilicity.

### 2.3. MoS_2_ Functionalization for Biological Applications

To improve the properties of MoS_2_ nanostructures for biological applications, various strategies for functionalization and surface modification have been developed. Commonly used approaches include both chemical and physical functionalization: chemical functionalization establishes new covalent bonds between the modifying agent and the substrate, while physical functionalization depends on interactions such as electrostatic attraction [72].

Following the synthesis of 2D MoS_2_, thin sheets are functionalized to specifically target biological molecules, making them suitable for biosensor development. Surface functionalization leads to charge displacement and the formation of surface dipoles, which can considerably modify the electrical band structure of 2D MoS_2_ [73]. Many studies have focused on functionalizing the basal plane of MoS_2_; however, challenges arise owing to the absence of dangling bonds and the extensive van der Waals surface area, which favors physisorption over stronger covalent or ionic bonding [74]. This results in molecules lying flat on the MoS_2_ surface, causing physisorption reactions that exhibit limited selectivity. However, by engineering the physisorbed layer, a selective surface for specific bioanalytes can be developed, thus enhancing the performance of biosensors.

Common approaches for modifying oxide and chalcogenide surfaces, such as silane and thiol-based techniques, are applicable to MoS_2_ as well. These methods work by creating hydroxyl and thiol groups on molybdenum that is exposed owing to sulfur deficiencies on the basal plane [75]. The efficacy of these functionalization techniques depends on the presence of these sulfur deficits to reveal the molybdenum sites.

Additionally, the edges of 2D MoS_2_ flakes, which can end with either metal or chalcogen atoms depending on the synthesis conditions, also present opportunities for functionalization [76]. Characteristics such as low-coordination step edges and kinks modify local surface effects and energies, which become more pronounced as the lateral dimensions decrease [77]. These features allow for the incorporation of functional groups, including hydroxyls and thiols, which can enhance bioactivity [78]. Moreover, disulfide bonds (R–S–S–C) can be used to attach organic molecules, such as proteins, while –SOH groups can promote interactions with proteins and carbohydrates.

Additional functionalization methods encompass in situ metal ion reduction, ring-opening polymerization, esterification, and free radical polymerization. According to Voiry et al., the covalent modification of metallic 1T MoS_2_ with amide and methyl groups induces a phase transition from the metallic 1T phase to the semiconducting 2H phase [79].

Gómez-Muñoz et al. proposed an innovative method for covalently coating chemically exfoliated MoS_2_ layers using functional polymer molecules [80]. This method uses a diazonium-anchored reaction, which facilitates the in situ radical polymerization of functional acrylate monomers, leading to the formation of a stable, homogeneous polymeric coating on the MoS_2_ surface. The incorporation of fluorinated acrylate monomers during functionalization improves the hydrophobicity and stability of MoS_2_, making it better suited for practical applications. This method not only simplifies the functionalization process but also ensures a high density of functional groups in the MoS_2_ layers. The polymer-coated MoS_2_ exhibited notable hydrophobic properties, with contact angles reaching 110° and 150° for different fluorinated acrylates. Additionally, polymer-coated MoS_2_ exhibited enhanced resistance to oxidation compared with unmodified MoS_2_. This functionalization approach not only enhances the material properties of MoS_2_ but also expands its potential applications in fields like optoelectronics, sensing, and catalysis, highlighting the versatility of 2D materials through targeted surface modification. Despite its advantages, this covalent coating method has certain limitations. This method relies on specific chemical conditions and reagents, such as diazonium salts, which may not be universally available and require careful handling because of their reactive nature.

Gan et al. explored the modification of MoS_2_ nanosheets with the organic molecule 6-(9H-carbazol-9-yl) hexane-1-thiol (CHT) to enhance their potential applications in nonlinear optics and optical limiting [81]. Although MoS_2_ is a 2D material known for its unique properties, its low reactivity and poor solubility in organic solvents present considerable challenges for its practical applications. The synthesis process involved dispersing MoS_2_ in N,N-dimethylformamide (and refluxing it with CHT, forming MoS_2_–CHT nanosheets. The spectroscopic analysis confirmed successful covalent bonding between MoS_2_ and CHT, which considerably improved MoS_2_’s solution processability. The modified MoS_2_–CHT materials exhibited enhanced nonlinear optical properties, particularly in the annealed MoS_2_–CHT/poly(methyl methacrylate) film. This film exhibited the highest nonlinear absorption coefficient and imaginary third-order susceptibilities at specific wavelengths. Structural changes during annealing, including a phase transition from the metallic 1T to the semiconducting 2H phase, contributed to these improvements. This study demonstrates that covalent functionalization effectively addresses MoS_2_’s solubility challenges, paving the way for the development of efficient optical limiters and other advanced photonic devices.

Functionality is also crucial for the stabilization of 2D MoS_2_ in ionic solutions, such as phosphate-buffered saline (PBS), which is commonly used in biosensor applications. Stable suspensions of MoS_2_ are critical for operation in these conditions [82]. Additionally, 2D MoS_2_ remains stable during photocatalytic reactions, allowing the use of optical methods for surface functionalization without compromising its properties.

The medical and sensor applications of metal and metal oxide NPs [83,84,85,86] have led to the development of synthetic methods for creating MoS_2_-based hybrids. The nanostructures of metals and metal oxides, known for their catalytic and electrical properties, are effectively embedded in MoS_2_ nanofilms. The supporting matrix of MoS_2_ provides uniform distribution and enhanced functionality of these nanostructures, resulting in improved conductivity and catalytic efficiency compared to unmodified MoS_2_ [87].

## 3. Plasmonic Nanomaterials

Plasmonic phenomena arise when light of an appropriate wavelength interacts with the surface of noble metals at a dielectric interface, thereby exciting conduction electrons and inducing their oscillation. This synchronized oscillation of electrons, known as plasmon resonance, generally occurs in particles approximately 300 nm in size or smaller [88,89]. Plasmonic nanomaterials, primarily made up of noble metals such as Au, Ag, and Cu, are highly valued for their unique optical characteristics, particularly in biological and medical fields [90,91,92,93]. These materials exhibit LSPR and surface-enhanced Raman scattering (SERS), which facilitate strong interactions with light at select wavelengths, resulting in enhanced electromagnetic fields in their vicinity. Such attributes pave the way for applications in imaging, sensing, and therapeutic delivery [94]. Au, Ag, and Cu are well suited for plasmonic applications because of their high charge–carrier concentrations and strong light absorption capabilities in the visible and NIR ranges. In contrast, 2D nanomaterials, owing to their atomic thinness and lower carrier concentrations, typically display low absorption in these wavelengths. For example, single-layer graphene absorbs only approximately 2.3% of incident light across the visible to mid-infrared (MIR) spectrum [95,96]. However, moderately doped graphene can absorb as much as 40% of incident light in the terahertz (THz) frequency range, facilitating efficient light–matter interactions and allowing for a tunable plasmonic response in this region [97].

In plasmonic sensors, Au, Ag, and Cu are frequently used owing to their strong effects in the visible and NIR regions. In LSPR-based sensors, variations in the local RI resulting from analyte binding led to a shift in the absorption peak, allowing for highly sensitive detection. Meanwhile, in SERS-based sensors, molecules adsorbed on the metal surface interact with the amplified electromagnetic field, resulting in enhanced molecular sensitivity.

While the plasmonic effects of 2D nanomaterials have been studied for LSPR sensor applications, their limited absorption in the visible and NIR regions restricts their direct use in plasmonic sensors. Therefore, 2D nanomaterials are often paired with metal nanostructures to enhance sensor performance. This enhancement is achieved through various mechanisms. (1) The high surface area and numerous functional groups in 2D nanomaterials provide many active sites for molecular binding, strengthening sensor interactions. (2) π–π stacking with biomolecules: 2D nanomaterials can interact with the aromatic rings of biomolecules such as proteins and nucleic acids, enhancing analyte immobilization. (3) Protection against oxidation: 2D materials such as graphene, hexagonal boron nitride, and TMDs can protect plasmonic metals from oxidation, increasing stability and sensor longevity. (4) Enhanced electric field and charge transfer: A 2D nanomaterial layer amplifies the electric field at the metal interface and facilitates charge transfer between the plasmonic metal and probe molecules owing to its high carrier mobility, further enhancing sensor sensitivity. These enhancement mechanisms establish plasmonic nanomaterials, particularly in combination with 2D materials, as effective tools for biological and medical sensing applications.

### 3.1. Localized Surface Plasmon Resonance

LSPR is a foundational phenomenon driving many of the functions of plasmonic nanomaterials [98,99]. This unique optical response can be accurately controlled by manipulating the dimensions, morphology, and composition of NPs, thereby dramatically enhancing sensitivity in sensing and imaging applications. In biological settings, LSPR is extensively used in biosensing to detect biomolecular interactions at extremely low concentrations [100,101]. Additionally, LSPR enhances contrast and resolution in imaging, facilitating the detailed observation of cellular processes [102,103]. Moreover, LSPR plays a crucial role in photothermal therapy (PTT), where NPs convert light into heat to selectively target and eliminate cancer cells while minimizing damage to adjacent healthy tissue [104].

The LSPR phenomenon originates from the collective oscillation of conduction band electrons in noble metal NPs, leading to enhanced optical absorption [105,106]. The plasmonic characteristics of NPs are highly influenced by factors such as shape, size, anisotropy, RI, and the interaction between metallic and dielectric components [107,108]. By fine-tuning these parameters, the LSPR absorption peak of NPs can be accurately controlled, rendering them suitable for various plasmonic applications. Various geometries, such as core@shell (CS), core/shell@shell (CSS), and decorated nanosheets, can be engineered to optimize optical properties. The properties of NPs are elucidated using a range of analytical approaches [109], including the finite element method [110], finite-difference time-domain theory [111,112], Mie theory [113,114], discrete dipole approximation [115], and effective medium theories [116,117].

### 3.2. Surface-Enhanced Raman Scattering

SERS is a key application of plasmonic nanomaterials, providing a powerful method for molecular detection and analysis [118,119]. This technique leverages the intensified electromagnetic fields generated by LSPR to dramatically amplify the Raman signals of molecules adsorbed onto NP surfaces [120,121]. Unlike conventional techniques that depend on fluorescent labeling, SERS enables the elucidation of molecular composition and structure without the need for tags [122]. This label-free approach minimizes interference and enhances analytical accuracy. Furthermore, SERS facilitates real-time monitoring of dynamic biological processes, providing valuable insights into molecular interactions and cellular activities [123].

## 4. MoS_2_–Plasmonic Hybrid Platforms

The integration of MoS_2_ with plasmonic materials in hybrid platforms has attracted considerable research attention [42,124]. Recent breakthroughs in the synthesis of these hybrid nanostructures have highlighted their synergistic properties and diverse applications [125,126]. Notably, it has been demonstrated that manipulating the number of MoS_2_ layers and the aggregate size of plasmonic NPs provides an effective strategy for tailoring the performance of TMD-based devices.

For instance, under both nonresonant and resonant excitation conditions, Nam et al. used PL spectroscopy, a widely used technique for evaluating device performance, to investigate the variations in electronic and optical properties based on the number of MoS_2_ layers and the dimensions of the gold nanoparticle (AuNP) aggregate [127]. They observed that the PL intensity in monolayer MoS_2_/AuNPs increased with larger aggregate sizes, irrespective of the excitation conditions. The strain induced by AuNPs leads to a red shift; however, as the aggregates increase in size, the influence of p-doping becomes more pronounced, resulting in a noticeable blue shift. In multilayer MoS_2_/AuNPs, a decrease in PL intensity is observed under nonresonant excitation, while an increase is observed with resonant excitation, primarily attributed to p-doping and LSPR, respectively. Notably, the change in spectral shape resulting from resonant excitation is only clearly discernible in small aggregates of AuNPs in all layers.

### 4.1. Synthesis of MoS_2_–Plasmonic Hybrid Platforms

A variety of methodologies are currently used to synthesize MoS_2_–plasmonic hybrid platforms (Table 1), primarily relying on seed-mediated growth techniques and controlled combination strategies of plasmonic materials with MoS_2_ nanostructures [128].

The majority of synthesis methods use MoS_2_ seeds, adopting bottom-up approaches. A promising strategy for high-yield synthesis is the nanosheet-templated epitaxial growth of nanostructures. Huang et al. developed a solution-based method that uses 2D MoS_2_ nanosheets to facilitate the epitaxial growth of Pd, Pt, and Ag nanostructures [129]. Their findings revealed that metal nanostructures on the MoS_2_ (001) surface predominantly exhibited (111) and (101) orientations.

Shi et al. proposed a controlled wet chemical approach to grow AuNPs on chemical vapor deposition (CVD)-grown MoS_2_ layers, demonstrating that the Au–MoS_2_ hybrids are primarily formed through non-covalent bonding [130]. The morphology, density, and size distribution of the metal NPs could be accurately controlled by manipulating defect densities in the MoS_2_ layers. Seed-mediated growth methods using MoS_2_ seeds considerably enhance the efficiency of plasmonic nanostructures, with MoS_2_ serving as a substrate for atomically distributed metal catalysts (ADMCs). In a systematic study, Shi et al. synthesized ADMCs by depositing non-noble single-atom metals onto chalcogen sites in MoS_2_ layers, resulting in a diverse range of stable ADMCs, including Pt, Pd, Rh, Cu, Pb, Bi, and Sn. This method underscores the broad applicability of ADMCs in heterogeneous catalytic systems [131].

**Table 1:** Advantages and limitations of synthesis methods for MoS_2_–plasmonic hybrid platforms.

**Methods**	**Advantages**	**Limitations**
CVD [128,130,132]	- High quality - Suitable for large-scale applications	- High equipment costs - Time-consuming
Hydrothermal synthesis [130]	- Eco-friendly - Cost-effective	- Requires precise temperature and pressure control - Time-consuming
Solution-based methods [130]	- Selective decoration of metal NPs - Allows processing at low temperatures	- Risk of nonuniform NP deposition - Limited scalability
Solution-phase epitaxial Growth [129]	- Controlled growth of noble metal structures - Well-defined interfaces	- Requires precise control of growth conditions - Potential for nonuniform growth
Site-specific electrodeposition [131]	- Precise control over catalyst placement	- Requires careful parameter optimization - Scalability challenges
In situ synthesis [133]	- Direct growth on MoS_2_ enhances interaction with substrates - Defects act as nucleation sites for improved dispersion	- Requires careful control over synthesis conditions - Potential variability in particle size and morphology
Mechanical exfoliation [129,130,131]	- Simple and reliable method for producing high-quality MoS_2_ - Effective for single-layer or few-layer materials	- Limited scalability for large-area applications - Labor-intensive with low yield
Liquid exfoliation [129,130,133]	- Scalable for large quantities - Can enhance material properties	- Results in mixtures of different sizes and thicknesses - Lower quality compared to CVD
Chemical etching [131,133]	- Facilitates selective material removal - Creates well-defined structures	- Involves hazardous chemicals - Difficult to control the etching process

Abbreviations: CVD, chemical vapor deposition; NP, nanoparticle.

In redox reactions, AuNPs can be synthesized using exfoliated MoS_2_ seeds as substrates for Au atom deposition [128,134]. MoS_2_ nanosheets exhibit remarkable reduction capabilities in HAuCl_4_ solutions, facilitating Au ion reduction and subsequent synthesis of Au/MoS_2_ nanostructures by controlling reactant ratios of Au ions and MoS_2_ seeds. Studies have demonstrated that defects in MoS_2_ promote the in situ synthesis of plasmonic Au nanocrystals, where MoS_2_ edges guide the epitaxial growth of these nanocrystals. The LaMer growth process and planar aggregation of Au/MoS_2_ nanoseeds enable the formation of Au atomic layers on heterostructures [133].

Defective sites on MoS_2_ also enhance the reduction of Au(III). Initial interactions between AuCl_4_^−^ and the MoS_2_ surface form a MoS_2_/AuCl_4_ redox pair, where Au(III) is reduced to AuNPs directly on MoS_2_ nanosheets [135]. Thus, MoS_2_ can serve as an electron donor in spontaneous redox reactions. Yuan et al. used atomic force microscopy to investigate Au(III) deposition on MoS_2_, revealing that photogenerated electrons from MoS_2_ under light irradiation considerably contributed to Au(III) reduction [136].

An alternative bottom-up strategy entails preparing metal NPs in advance and using them as templates for hybrid fabrication. Among these methods, CVD is commonly used for the epitaxial growth of MoS_2_ layers [137]. CVD provides control over the thickness and morphology of MoS_2_ nanostructures, as exemplified by Yang et al., who synthesized monolayer MoS_2_ ribbons on high-Miller-index Au facet templates [132].

In another study, prepatterned Au seeds served as nucleation sites for MoS_2_ monolayers, enabling geometric control via CVD. The seeding effect, resulting from the favorable formation energy of MoS_2_ on Au surfaces, directed the growth of MoS_2_ monolayers at these specific locations [138,139].

Shi et al. devised a novel technique to selectively grow AgNPs on atomically layered MoS_2_ under continuous 808 nm laser irradiation. This laser-induced growth is driven by photothermal conversion rather than direct laser energy, with AgNPs enhancing MoS_2_’s thermal storage and stability owing to the surface plasmon resonance properties of silver [140].

Garoli et al. proposed a reliable approach for fabricating hybrid plasmonic nanopores through the controlled deposition of MoS_2_ flakes onto metallic apertures. This configuration offers localized plasmonic enhancement in the 2D nanopore, rendering it suitable for single-molecule sensing and sequencing in flow-through systems. Metallic NPs can further intensify electromagnetic field confinement by synchronizing their resonance with that of the nanopore, paving the way for solid-state nanopores with integrated plasmonic capabilities [141].

Zuo et al. introduced a groundbreaking technique for the non-thermal induction of photogenerated electrons in MoS_2_, facilitating interactions with Ag and Pt metal cations [142]. These interactions lead to the reduction and subsequent in situ deposition of metal NPs onto MoS_2_ nanosheets, forming metal–MoS_2_ nanohybrids. Notably, this method requires only metal salts to provide the required cations, eliminating the need for additional chemical reagents and reducing the risk of introducing toxic byproducts or environmental contaminants. The characterization techniques used confirmed the formation of highly crystalline metal NPs on the MoS_2_ nanosheets, demonstrating the doping effects of the metal NPs and the modifications made to the MoS_2_ structure. Moreover, the Ag–MoS_2_ hybrids exhibited exceptional SERS activity, with an enhancement factor of 1.32 × 10^7^ and a detection limit as low as 10^−11^ M, indicating considerable potential for sensing chemical and biological molecules. Additionally, the Pt–MoS_2_ hybrids exhibited excellent HER performance, characterized by a low Tafel slope of 25 mV/decade and a TOF of 11.15 H_2_ s^−1^ at 220 mV, highlighting their promising applications in future hydrogen production.

### 4.2. Biological Applications of MoS_2_–Plasmonic Hybrid Platforms

#### 4.2.1. MoS_2_–Plasmonic Hybrid Platforms for Biosensing

The integration of 2D materials with plasmonic structures has opened promising avenues for various cutting-edge biomedical applications (Table 2). MoS_2_, known for its exceptional electronic properties, extensive surface area, and biocompatibility, is ideally suited for biosensing applications. By combining MoS_2_ with plasmonic materials that exhibit LSPR, these hybrid platforms can dramatically enhance signal detection and sensitivity.

**Figure 4:**
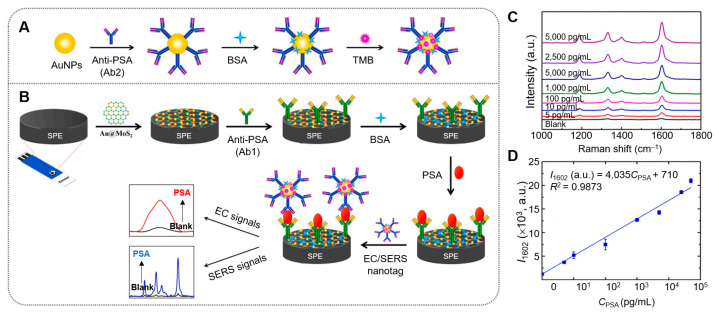
(**A**) Schematic illustration of nanotag preparation (TMB/Ab2/AuNPs) and (**B**) the construction process of the EC/SERS dual-mode immunosensor. (**C**) SERS spectra of PSA samples at varying concentrations in PBS and (**D**) the corresponding plot of PSA concentrations versus SERS intensities at the Raman shift of 1602 cm^−1^ in PBS. Reprinted with permission from Ref. [143]. Copyright (2024) Elsevier B.V.

Yaiwong et al. devised a dual-mode immunosensor that combines SERS with electrochemical (EC) techniques to detect prostate-specific antigen (PSA), a biomarker associated with prostate cancer [143] (Figure 4). This sensor uses a hybrid of AuNPs on 2D MoS_2_, modified on the working carbon electrode of an SPE. A primary antibody (Ab1) is immobilized on the modified electrode, forming an Ab1/Au@MoS_2_/SPE structure for specific PSA recognition. Simultaneously, AuNPs are conjugated with a secondary antibody (Ab2) and the probe molecule 3,3′,5,5′-tetramethylbenzidine (TMB), creating nanotags (TMB/Ab2/AuNPs) that exhibit strong SERS and EC responses. When PSA is present, sandwich immunocomplexes form through antigen–antibody interactions (Ab1–PSA–Ab2). Differential pulse voltammetry is used for EC detection, while SERS data are acquired with a handheld Raman spectrometer using a 785 nm laser. This system demonstrates high selectivity and sensitivity, with limits of detection (LODs) of 3.58 nM for EC and 4.83 nM for SERS, enabling the effective quantification of PSA in human serum samples, suggesting its potential as an alternative tool for early cancer diagnostics.

Liu et al. synthesized nonmetallic plasmonic MoS_2_ nanosheets via a hydrothermal top-down method to enhance the electrochemiluminescence (ECL) signal of sulfur-doped boron nitride quantum dots (S–BN QDs) [144]. However, close or far spacing between ECL luminophores and plasmonic NPs was observed to reduce the ECL intensity of both the nano-luminophore and the plasmonic NPs. This innovative DNA sensor achieved high detection efficiency for the hepatitis C virus gene, with a detection range of 0.5 pM to 1 nM and an LOD of 0.17 pM. Nor et al. developed a rapid and highly sensitive SPR detection platform for the SARS-CoV-2 spike protein (*S* protein) by utilizing various support layers, including MoS_2_–COOH, 3-mercaptopropionic acid (3-MPA), and self-assembled monolayers of 11-mercaptoundecanoic acid (11-MUA) [145]. Among these, the SPR setup incorporating the MoS_2_–COOH layer demonstrated the highest sensitivity of 203.55°/RIU, significantly outperforming 11-MUA (89.89°/RIU) and 3-MPA (108.90°/RIU). The exceptional sensitivity of the MoS_2_–COOH layer can be attributed to a combination of factors. First, MoS_2_–COOH exhibits high chemical stability and strong resistance to oxidation, ensuring consistent and reliable interactions at the sensor surface. Additionally, MoS_2_–COOH has a large surface area, which enhances the immobilization of biomolecules, thus strengthening the binding interactions with the SARS-CoV-2 spike protein. Furthermore, the presence of carboxylic acid functional groups in MoS_2_–COOH improves the bioaffinity of the sensor surface, enabling stronger covalent bonding with immobilized ligands. Moreover, MoS_2_’s unique optical properties amplify the surface plasmon resonance effect, leading to more pronounced shifts in resonance angle when the target binds to the sensor surface. The specialized architecture of the MoS_2_–COOH layer further optimizes the light coupling and plasmonic effects, thereby enhancing the overall detection capability. Due to these combined properties, the MoS_2_–COOH layer considerably outperforms traditional thiol-based self-assembled monolayers, making it an ideal candidate for developing highly sensitive SPR biosensors for virus detection. All configurations exhibited outstanding sensitivity, excellent linearity, and low detection limits in the ng/mL range, establishing a rapid, noninvasive, and ultrasensitive SPR detection platform for detecting viral pathogens at low concentrations.

#### 4.2.2. MoS_2_–Plasmonic Hybrid Platforms for Bioimaging

MoS_2_–plasmonic hybrid platforms are extensively used in bioimaging applications, facilitating the investigation of cellular and tissue properties, as well as the real-time monitoring of intracellular NP and drug trafficking. Although bioimaging encompasses various imaging modalities, such as light [155,156], X-rays [157,158], electron microscopy [159], positron emission [160,161], and magnetic resonance [162,163], this review will focus only on bioimaging studies using optical microscopy.

**Figure 5:**
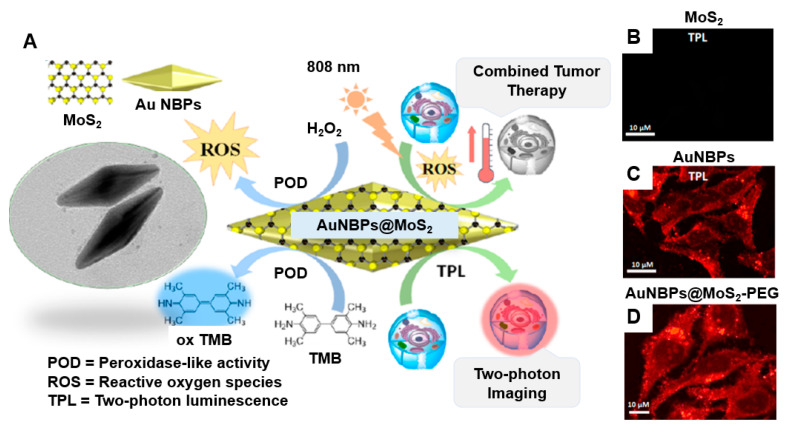
(**A**) Two-photon cell imaging using AuNBPs@MoS_2_. The two-photon confocal laser scanning microscopy images show HeLa cancer cells incubated with (**B**) MoS_2_, (**C**) AuNBPs, and (**D**) AuNBPs@MoS_2_–PEG (50 μg/mL) for 12 h. Reprinted with permission from Ref. [146]. Copyright (2018) American Chemical Society.

Maji et al. [146] developed a hybrid structure that combines gold nanobipyramids (AuNBPs) with a MoS_2_ semiconductor layer, creating a multifunctional tool for anticancer therapy and two-photon bioimaging. This hybrid material showed considerably improved LSPR properties when excited owing to its anisotropic structure and the high electron density of MoS_2_. As a result, there was an increased in situ photogeneration of reactive oxygen species (ROS), including singlet oxygen (^1^O_2_) and hydroxyl radicals (•OH) (Figure 5A). The combined effects of enhanced photothermal conversion and ROS generation led to markedly improved anticancer efficacy of the AuNBPs@MoS_2_ hybrid. An increase in optical absorbance, leading to enhanced photothermal activity (from 25 °C to 60.3 °C), was achieved owing to the strong electronic connection and plasmonic coupling between the AuNPs and MoS_2_ nanosheets. Importantly, the AuNBPs@MoS_2_ hybrid demonstrated peroxidase-like nanozyme activity under acidic conditions, with its performance doubling in the presence of NIR light, which promoted additional ROS generation through plasmonic effects. The subcellular localization of AuNBPs@MoS_2_ was confirmed through in vitro cellular uptake studies in HeLa cells, revealing clear two-photon luminescence (TPL) imaging. These catalytic and photothermal properties were effectively used to achieve high therapeutic efficacy against HeLa cancer cells, resulting in cell viability of 13.2%, highlighting the potential of sequential and combinatorial therapy approaches. The successful internalization of AuNBPs@MoS_2_ in cancer cells, validated through TPL imaging, facilitated concurrent anticancer therapies via catalytic and photothermal mechanisms (Figure 5B–D).

Xu et al. [147] developed a composite nanoprobe (Ag@MoS_2_) for in situ fluorescence imaging and quantitative analysis of intracellular adenosine triphosphate (ATP) levels in HeLa cells using fluorescence spectrometry and inductively coupled plasma mass spectrometry (ICP–MS). This probe was created by adsorbing DNA–Ag nanoclusters (AgNCs) onto MoS_2_ nanosheets, with the DNA–AgNCs being synthesized using an ATP aptamer as a template. Initially, the fluorescence of the DNA–AgNCs was quenched by MoS_2_. Upon incubation with cells, intracellular ATP recognized the aptamer sequence, resulting in the detachment of the DNA–AgNCs from the MoS_2_ nanosheets and restoring fluorescence. Here, AgNCs acted as both a fluorescent label for imaging and an elemental tag for quantifying intracellular ATP by detecting ^107^Ag via ICP–MS. The measured ATP concentration in HeLa cells using this method was 24.6 ± 1.7 nM, closely aligning with the ATP test kit result of 20.4 ± 0.8 nM.

#### 4.2.3. MoS_2_–Plasmonic Hybrid Platforms for Phototherapy

Phototherapy, encompassing PTT and photodynamic therapy (PDT), is gaining prominence as an alternative to traditional cancer treatments [164,165]. Unlike conventional therapies, phototherapy offers unique advantages: accurate control over irradiation parameters (location, duration, and intensity). This section explores the use of MoS_2_–plasmonic hybrid platforms in both PTT and PDT.

In PTT, a phototransducer material efficiently absorbs light, especially in the NIR range, and converts it into heat. This localized thermal ablation targets cancerous cells or tissues [166,167]. NIR light is particularly advantageous for PTT owing to its deep tissue penetration, low absorption by biological tissues, and noninvasive nature, making PTT a promising therapeutic option for treating tumors [165,168]. Various organic NIR-responsive dyes and nanomaterials have been investigated as potential phototransducer materials.

Among nanomaterials, MoS_2_ stands out for its unique properties: catalytic activity, direct bandgap, broad spectral response, and high photothermal conversion efficiency (PCE). These attributes make it a key material in photocatalysis research [153,169]. Notably, MoS_2_ has gained recognition as a biocompatible nanomaterial with exceptional light-to-heat conversion capabilities. While pure MoS_2_ exhibits promising biocompatibility, its catalytic and antibacterial performance may be limited. To address this, researchers have explored the combination of MoS_2_ with plasmonic metal NPs, showing great promise for enhancing the efficacy of phototherapy [170]. This performance enhancement can be primarily attributed to the LSPR phenomenon, which is exhibited by Au plasmonic NPs. This phenomenon amplifies local electromagnetic fields and improves light absorption. The interaction between AuNPs and MoS_2_ generates high-energy “hot electrons”, which are effectively transferred to the MoS_2_ nanomaterial, injecting additional energy into the system. Moreover, MoS_2_ possesses inherent piezoelectric properties that enable it to generate an electric field when subjected to mechanical strain, further facilitating the separation and migration of charge carriers. The synergy between LSPR and piezophototronics considerably reduces the recombination rates of electron–hole pairs, which, in turn, improves the catalytic activity of MoS_2_. This synergy not only enhances charge dynamics and light absorption but also results in more efficient PTT and PDT, yielding improved outcomes in various applications, such as cancer treatment and microbial disinfection.

Liu et al. engineered a Bi/MoS_2_ heterojunction complex in a fibrin gel, which demonstrated antibacterial efficiencies of 99.2% and 99.7% against *S. aureus* and *E. coli*, respectively, under 808 nm NIR irradiation, surpassing the MoS_2_-only group [148]. This enhanced antibacterial effect stems from the synergistic action of photothermal conversion and ROS production upon light exposure. This improvement in antibacterial efficacy is mainly due to the LSPR phenomenon exhibited by the Bi NPs. Under 808 nm NIR light, the electrons on the surface of the Bi NPs resonate, generating an amplified electromagnetic field. This resonance leads to enhanced light absorption, resulting in localized heating that considerably improves the photothermal conversion efficiency, surpassing that of MoS_2_ alone. In addition, the inclusion of Bi NPs improves charge separation and transport within the heterojunction, which is crucial for ROS generation under NIR irradiation. The synergistic effects of intense localized heating generated by the Bi NPs and ROS produced by MoS_2_ disrupt bacterial membranes and induce oxidative damage. This dual mechanism explains the high antibacterial activities of 99.2% and 99.7% against *S. aureus* and *E. coli*, respectively. These findings highlight the potential of the Bi/MoS_2_ heterojunction complex as a highly effective antimicrobial agent, particularly for wound healing applications. Furthermore, the photothermal effect induced protein denaturation, effectively eradicating bacteria in both in vitro and in vivo settings. In vivo animal studies corroborated the bactericidal capabilities of the gel under light irradiation while simultaneously demonstrating its potential in promoting diabetic wound healing through the stimulation of collagen formation, re-epithelialization, and angiogenesis.

**Figure 6:**
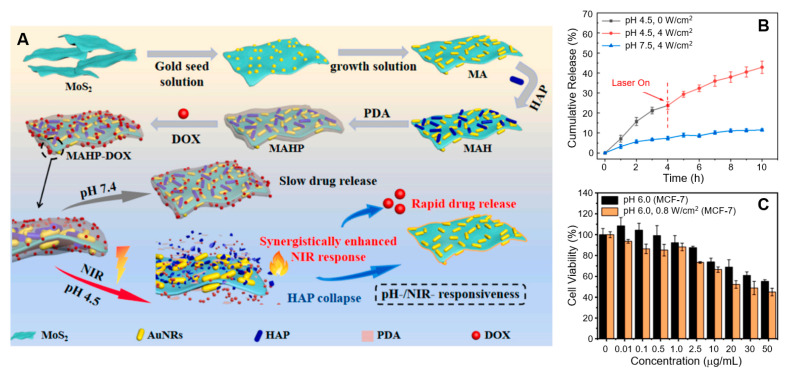
(**A**) Preparation process of MAHP and its drug release mechanism under a synergistic photothermal effect. (**B**) DOX release from MAHP under varying pH conditions and NIR laser irradiation. (**C**) Cytotoxicity assessment of MAHP–DOX in MCF-7 cells with and without laser irradiation at pH 6.0. Reprinted with permission from Ref. [149]. Copyright (2023) Elsevier B.V.

Wang et al. used an in situ growth technique to integrate Au NRs with MoS_2_, synthesizing MoS_2_@AuNRs (MA) NPs with an impressive PCE of 42.16% [149]. Subsequently, hydroxyapatite (HAP) and polydopamine (PDA) were self-assembled onto the MoS_2_@AuNRs NPs (Figure 6A). These NPs exhibited substantial temperature increases, reaching 34.1 °C for MoS_2_@AuNRs and 30 °C for MoS_2_@AuNRs/HAP/PDA (MAHP) after 60 min of laser irradiation. These findings corroborate the potent NIR-dependent photothermal properties of MoS_2_@AuNRs, with temperature increases directly proportional to NP concentration and exposure time. To evaluate the NIR and pH-responsive drug release performance of MAHP, 4 W/cm^2^ NIR laser irradiation was applied after 4 h at pH 4.5 (Figure 6B). The release of the anticancer drug doxorubicin hydrochloride (DOX) was limited to 26.57% after 4 h and reached 49.95% after 10 h. This phenomenon is attributed to the heat generated by the MA NPs, which accelerates the dissociation of the weak electrostatic bond between MAHP and DOX. Notably, the MoS_2_@AuNRs/HAP/PDA NPs exhibited a high DOX loading capacity of 80%, along with pH- and NIR-responsive drug release properties. This is facilitated by the structural breakdown of HAP in acidic conditions and the strong NIR response of MoS_2_@AuNRs. In vitro cell viability assays demonstrated that MoS_2_@AuNRs/HAP/PDA NPs effectively inhibited MCF-7 cells under NIR laser irradiation and acidic conditions (Figure 6C).

In PDT, a photosensitizer (PS) is used to generate ROS upon light exposure [171]. The PS transitions to an excited state, transferring energy to oxygen molecules in its vicinity, leading to ROS formation. These highly reactive species oxidize lipids, DNA, and proteins, ultimately causing cell death [172,173]. PDT offers minimally invasive treatment and effectively targets even small, hidden tumor cells.

Li et al. developed MoS_2_–AuNRs–aptamer NPs capable of actively targeting lipopolysaccharides (LPS) on Gram-negative bacteria, enabling effective antimicrobial action against multidrug-resistant *Pseudomonas aeruginosa* (MRPA) in wound models [150]. These targeted NRs exhibited superior antimicrobial efficacy compared to nontargeted PTT, effectively eliminating MRPA bacteria through physical damage. The enhanced effectiveness of the MoS_2_–AuNRs–aptamer is primarily attributed to its ability to actively target LPS on the MRPA surface. This targeted approach facilitates more efficient delivery of PTT directly to the bacterial cells, enabling localized heating and physical disruption of the bacterial structure under NIR irradiation. The aptamer’s specific binding to LPS facilitates NP accumulation at the infection site and considerably enhances thermal damage to the bacteria. As a result, the targeted approach acheives a more pronounced reduction in bacterial viability than nontargeted PTT, which lacks specificity and risks damaging surrounding healthy tissues without effectively targeting the bacteria. Furthermore, the use of targeted NPs substantially reduced M1 inflammatory macrophages, thereby promoting an improved wound healing environment.

Younis et al. combined MoS_2_ and AuNRs to create a dual plasmonic PTT nanoagent, achieving a remarkable temperature increase of up to 60 °C with a PCE of 68.8% in 5 min under low-power NIR laser irradiation (0.2 W/cm^2^) [45]. This enhanced PCE and PTT efficacy were attributed to the synergistic plasmonic PTT (PPTT) effects of the dual plasmonic agents (MoS_2_ and AuNRs), enabling the efficient release of electrostatically bound indocyanine green (ICG) for simultaneous PDT and PPTT. AuNRs exhibit strong LSPR, efficiently absorbing NIR light and converting it into heat, while MoS_2_ contributes with its large surface area and NIR light absorption abilities, which aid in charge separation. The combination of these materials enhances light absorption and increases the overall photothermal conversion efficiency, resulting in a substantial temperature rise of up to 60 °C under low-power laser irradiation. This synergistic effect improves heat generation and promotes the efficient thermal release of electrostatically bound ICG, thereby activating PDT and PPTT simulatenously, ultimately delivering superior therapeutic outcomes in cancer treatment.

Yougbare et al. synthesized light-responsive hybrids that have attracted considerable attention in the biomedical field for antibacterial applications [151]. Specifically, they developed metallic molybdenum disulfide nanosheets (1T-MoS_2_ NSs) activated by visible light and combined them with plasmonic AuNRs exhibiting absorption at a wavelength of 808 nm. The AuNRs were successfully prepared via electrostatic adsorption onto 1T-MoS_2_ NSs forming MoS_2_@AuNRs for potential phototherapy applications. The authors demonstrated that the solution temperature MoS_2_@AuNR increased from 25 °C to 66.7 °C within 10 min of NIR laser irradiation at 808 nm, emphasizing the effectiveness of the photothermal effect. In addition, the MoS_2_@AuNR hybrids generated RSO under visible light irradiation, contributing to their photodynamic effects. The antibacterial efficacy of MoS_2_@AuNRs against *E. coli* was confirmed using agar plate count assays, illustrating their potential use in phototherapy. Notably, the combination of PTT and PDT provided by the MoS_2_@AuNR hybrids demonstrated greater antibacterial activity compared with individual therapy. This remarkable synergistic effect highlights the innovative potential of light-activated MoS_2_@AuNR hybrids to combat bacterial infections.

Liu et al. developed a novel PTT/PDT antitumor theranostic nanoplatform based on chlorin e6 (Ce6)-loaded PEG–MoS_2_–AuNP hybrids. These hybrids substantially enhanced PTT efficacy for cancer treatment under 808 nm laser irradiation, attributed to their unique material properties and synergistic mechanisms. The MoS_2_ nanomaterial provides strong NIR absorption, while the AuNPs enhance photothermal conversion through SPR. Together, these components generate substantial heat under laser exposure, achieving temperatures sufficient to induce thermal damage to cancer cells. Additionally, the hyperthermic environment promotes the release of the Ce6 photosensitizer, which activates PDT by generating ROS upon irradiation. This dual-action approach directly destroys tumor cells via thermal effects and enhances PDT efficacy, creating a synergistic effect that markedly improves therapeutic outcomes against tumors. Furthermore, Ce6 release from the hybrids produced strong NIR fluorescence signals, initiating the PDT effect for antitumor therapy. Notably, PTT effectively triggered both Ce6 release and ROS generation, resulting in a synergistic PTT–PDT effect that significantly improved cancer treatment efficacy [152].

Wei et al. developed an artificial nanozyme, Fe_3_O_4_@MoS_2_–Ag, using a two-step hydrothermal method involving the in situ growth of AgNPs [153]. This nanozyme exhibited remarkable antibacterial activity against *E. coli*, achieving approximately 69.4% inhibition through the generation of ROS and the release of silver ion (Ag^+^) ions. Notably, its disinfection efficacy was further enhanced by the NIR photothermal properties of Fe_3_O_4_@MoS_2_–Ag, reaching nearly 100% inhibition. The antibacterial mechanism relies on the synergistic effects of photothermal heating, peroxidase-like activity, and Ag^+^ ions release. The Fe_3_O_4_@MoS_2_–Ag nanozyme exhibits peroxidase-like activity and catalyzes hydrogen peroxide conversion into ROSs—such as hydroxyl radicals—that effectively damage bacterial membranes. Concurrently, Ag^+^ release disrupts bacterial cellular functions by permeating membranes and interfering with vital processes. Additionally, the nanozyme’s photothermal properties allow it to absorb NIR light, generating localized heat that enhances ROS activity and promotes Ag^+^ release. This combination of mechanisms—where heat increases membrane permeability and ROS and Ag^+^ ions exert their antibacterial effects—results in considerably enhanced antibacterial efficacy, demonstrating the effective synergy of these three components in combating bacterial infections. The defect-rich rough MoS_2_ surface facilitates bacterial capture, enabling the accurate and rapid action of ^•^OH and Ag^+^ on the *E. coli* membrane, supported by local hyperthermia. This approach demonstrated broad-spectrum antibacterial efficacy against Gram-positive and Gram-negative bacteria, fungal pathogens, and drug-resistant strains.

Rodriguez-da-Silva et al. synthesized MoS_2_ NFs through a hydrothermal method and used them as substrates for the deposition of small spherical monometallic Au or bimetallic AuAg NPs [154]. These hybrid nanostructures are novel photothermal-assisted catalysts, exhibiting enhanced catalytic efficiency under NIR laser irradiation. The improved catalytic performance results from the synergistic effect of combining MoS_2_ NFs with either bimetallic AuAg or monometallic AuNPs. This combination facilitates electron transfer during the reduction of 4-nitrophenol to 4-aminophenol. MoS_2_ serves as a 2D platform that absorbs light energy and converts it into thermal energy, thereby enhancing catalytic activity. The alloyed AuAg NPs increase electron density at the gold active sites, with silver donating electron density to adjacent gold atoms, thus increasing their reactivity. Additionally, MoS_2_’s strong NIR light absorption enables efficient conversion of this energy into heat, which accelerates reaction kinetics. The enhanced kinetics creates a cooperative catalytic mechanism where MoS_2_ provides photothermal effects and acts as a substrate, while the metal NPs engage in electron transfer and reduction reactions. This synergistic interaction substantially enhances the catalytic process, outperforming the independent performance of each component. Notably, the synthesized hybrid NPs integrate these critical factors, considerably accelerating the reduction rate of 4-nitrophenol to 4-aminophenol.

## 5. Challenges and Future Perspectives

MoS_2_–plasmonic hybrid platforms have demonstrated considerable potential in various biological applications, including biosensing, bioimaging, and phototherapy [33,42,87,174]. However, to fully realize their clinical potential, several challenges must be addressed, including synthesis reproducibility, toxicity, stability under physiological conditions, targeted delivery, and scalable manufacturing.

The inherent reactivity of MoS_2_ can lead to degradation or alteration of its optical and electrical properties over time, potentially compromising the functionality of the hybrid system. To mitigate this, advanced encapsulation and passivation techniques must be developed to protect MoS_2_ and maintain the overall stability of the hybrid structure.

Future research should focus on developing robust encapsulation strategies, such as employing biocompatible polymers or inorganic coatings like silica or alumina, to shield MoS_2_ from degradation while preserving its optical and electrical properties. in addition, in vivo studies are essential to assess the long-term effects of these encapsulation methods on the stability and performance of hybrid platforms under dynamic physiological conditions.

Improving biocompatibility and in vivo performance also necessitates thorough toxicological studies to ensure minimal adverse effects on healthy tissues. Concurrently, designing and synthesizing targeted delivery systems, such as ligand-conjugated NPs or aptamer-functionalized platforms, are crucial for ensuring the specific delivery of hybrids to diseased tissues while minimizing off-target accumulation and side effects.

Synthesis reproducibility is another notable challenge. Methods to consistently produce hybrid structures with uniform size, shape, and composition—such as microfluidic or continuous flow techniques—are essential for clinical translation. Investigating the effects of synthesis parameters, such as temperature, reactant concentration, and reaction time, on the physicochemical properties and biological performance of the platforms is necessary. Addressing variability in synthesis is critical to ensuring consistent performance and safety.

Navigating the regulatory landscape for clinical translation is also vital. The complexity of MoS_2_–plasmonic hybrids may pose challenges in meeting regulatory requirements, potentially delaying approval and clinical use. Clear guidelines are needed to facilitate the translation of these innovative therapies into clinical practice.

To fully exploit the potential of MoS_2_–plasmonic hybrid platforms, enhancing their sensitivity and specificity is crucial. Research should focus on optimizing the size, morphology, and composition of plasmonic components to maximize optical signal enhancement, thereby achieving enhanced detection and imaging capabilities.

Finally, practical devices for real-life applications must be developed. Efforts should be directed toward integrating MoS_2_–plasmonic hybrid platforms into user-friendly, portable biosensors or point-of-care devices for real-time diagnostics and personalized medicine.

Addressing these challenges requires a multidisciplinary approach involving close collaboration among materials scientists, engineers, biologists, and clinicians. With continued research and development, MoS_2_–plasmonic hybrid platforms can revolutionize diagnostics, therapeutics, and personalized healthcare.

## 6. Conclusions

MoS_2_–plasmonic hybrid platforms are revolutionizing nanomedicine by merging the extraordinary optical, electrical, and catalytic properties of MoS_2_ with the signal-enhancing capabilities of plasmonic materials. These hybrids have emerged as exceptional tools for biosensing, bioimaging, and phototherapy, offering accurate and minimally invasive solutions for diagnostics and treatments. The synergistic interactions between MoS_2_ and plasmonic NPs enable advancements in sensitivity, specificity, and therapeutic efficacy, making them highly versatile for a wide range of biomedical applications.

Although MoS_2_–plasmonic hybrid platforms have demonstrated potential in various biological applications, challenges such as synthesis reproducibility, toxicity, stability under physiological conditions, targeted delivery, and scalable manufacturing remain. Advances in synthesis techniques and the fostering of interdisciplinary collaboration will play a crucial role in overcoming these limitations. With sustained innovation, MoS_2_–plasmonic hybrid platforms are poised to revolutionize biomedical science by providing next-generation solutions for complex health challenges. These platforms have the potential to redefine diagnostic and therapeutic paradigms, paving the way for groundbreaking advancements in personalized healthcare.

## Figures and Tables

**Table 2:** Biological applications of MoS_2_–plasmonic hybrid platforms.

**Hybrid Platform**	**Application**	**Target**
AuNPs on 2D MoS_2_ modified on the working carbon electrode of an SPE [143]	Detection of PSA, a biomarker associated with prostate cancer	PSA
Nonmetallic plasmonic MoS_2_ nanosheets [144]	DNA sensor for detecting the hepatitis C virus gene	Hepatitis C virus gene
Carboxyl-functionalized MoS_2_ (MoS_2_–COOH) in an SPR detection platform [145]	Detection of the SARS-CoV-2 spike protein (*S* protein)	SARS-CoV-2 spike protein
AuNBPs combined with a MoS_2_ semiconductor layer (AuNBPs@MoS_2_) [146]	Anticancer therapy	HeLa cancer cells
Composite nanoprobe (Ag@MoS_2_) comprising DNA–Ag nanoclusters adsorbed onto MoS_2_ nanosheets [147]	In situ fluorescence imaging and quantitative analysis of intracellular ATP levels	Intracellular ATP in HeLa cells
Bi/MoS_2_ heterojunction complex in a fibrin gel [148]	Antibacterial treatment for diabetic wounds	*S. aureus* and *E. coli* bacteria
MA with HAP and PDA [149]	Controlled drug release	Anticancer drug DOX and MCF-7 cancer cells
MoS_2_–AuNRs–aptamer NPs [150]	Selective photothermal therapy for antimicrobial action	MRPA
MoS_2_ and AuNRs dual PPTT nanoagent [45]	Synergistic cancer phototherapy	ICG for cancer treatment
MoS_2_@AuNRs (1T-MoS_2_ nanosheets with AuNRs) [151]	Synergistic photothermal and photodynamic antibacterial therapy	*E. coli* bacteria
PEG–MoS_2_–AuNP hybrids loaded with Ce6 [152]	Stepwise photothermal and photodynamic therapy for cancer treatment	Tumor cells
Fe_3_O_4_@MoS_2_–Ag nanozyme [153]	Enhanced antibacterial therapy	*E. coli* bacteria
NFs with AuAg alloyed NPs [154]	Photothermal-assisted catalytic reaction	Reduction of 4-NiP to 4-AP

Abbreviations: ATP, adenosine triphosphate; PSA, prostate-specific antigen; SPE, screen-printed electrode; AuNBPs, gold nanobipyramids; AuNRs, gold nanorods; DOX, doxorubicin hydrochloride; MA, MoS_2_@AuNRs nanoparticles; HAP, hydroxyapatite; PDA, polydopamine; MRPA, multidrug-resistant pseudomonas aeruginosa; ICG, indocyanine green; PPTT, plasmonic photothermal therapy; Ce6, chlorin e6; NFs, MoS_2_ nanoflowers; NiP, nitrophenol; AP, aminophenol; SARS-CoV-2, severe acute respiratory syndrome coronavirus 2; HeLa, Henrietta Lacks; MCF-7, Michigan Cancer Foundation-7; NP, nanoparticle.

## References

[1] Hou J., Hu C., Li H., Liu H., Xiang Y., Wu G., Li Y. (2025). Nanomaterial-Based Magnetic Solid-Phase Extraction in Pharmaceutical and Biomedical Analysis. J. Pharm. Biomed. Anal..

[2] Pandey M., Nazar R., Elella M.H.A., Praharaj S., Makhado E., Rout D., Gilani E.H. (2024). A Comprehensive Review of Recent Developments in Biomedical Materials Based on Graphene-Modified Bio-Nanocomposites. BioNanoScience.

[3] Wang L., Ji Y., Chen Y., Zheng S., Wang F., Li C. (2024). Recent Research Progress of Fluorescence Biosensors Based on Carbon Dots in Early Diagnosis of Diseases. TrAC Trends Anal. Chem..

[4] Yao S., Wang Y., Mou X., Yang X., Cai Y. (2024). Recent Advances of Photoresponsive Nanomaterials for Diagnosis and Treatment of Acute Kidney Injury. J. Nanobiotechnol..

[5] Chen S., Xie Y., Ma K., Wei Z., Ran X., Fu X., Zhang C., Zhao C. (2024). Electrospun Nanofibrous Membranes Meet Antibacterial Nanomaterials: From Preparation Strategies to Biomedical Applications. Bioact. Mater..

[6] Nam N.N., Trinh T.N.D., Do H.D.K., Phan T.B., Trinh K.T.L., Lee N.Y. (2025). Advances and Opportunities of Luminescence Nanomaterial for Bioanalysis and Diagnostics. Spectrochim. Acta A Mol. Biomol. Spectrosc..

[7] Niazi S., Khan I.M., Akhtar W., ul Haq F., Pasha I., Khan M.K.I., Mohsin A., Ahmad S., Zhang Y., Wang Z. (2024). Aptamer Functionalized Gold Nanoclusters as an Emerging Nanoprobe in Biosensing, Diagnostic, Catalysis and Bioimaging. Talanta.

[8] Castro K.P.R., Colombo R.N.P., Iost R.M., da Silva B.G.R., Crespilho F.N. (2023). Low-Dimensionality Carbon-Based Biosensors: The New Era of Emerging Technologies in Bioanalytical Chemistry. Anal. Bioanal. Chem..

[9] Prashanth G.K., Dileep M.S., Gadewar M., Ghosh M.K., Rao S., Giresha A.S., Prashanth P.A., Swamy M.M., Yatish K.V., Mutthuraju M. (2024). Zinc Oxide Nanostructures: Illuminating the Potential in Biomedical Applications: A Brief Overview. BioNanoScience.

[10] Adul-Rasool A.A., Athair D.M., Zaidan H.K., Rheima A.M., Al-Sharify Z.T., Mohammed S.H., Kianfar E. (2024). 0,1,2,3D Nanostructures, Types of Bulk Nanostructured Materials, and Drug Nanocrystals: An Overview. Cancer Treat. Res. Commun..

[11] Rafiei-Sarmazdeh Z., Morteza Zahedi-Dizaji S., Kafi Kang A., Ameen S., Akhtar M.S., Shin H.-S. (2020). Two-Dimensional Nanomaterials. Nanostructures.

[12] Preethi S., Varghese S., Biswas K., Vijayalakshmi N. (2024). Unveiling the Properties of Layered 2D-Based Nano-Material Flexible Electronics in Biomedical Applications: A Review. J. Mater. Sci..

[13] Maghimaa M., Sagadevan S., Boojhana E., Fatimah I., Lett J.A., Moharana S., Garg S., Al-Anber M.A. (2024). Enhancing Biocompatibility and Functionality: Carbon Nanotube-Polymer Nanocomposites for Improved Biomedical Applications. J. Drug Deliv. Sci. Technol..

[14] Novoselov K.S., Geim A.K., Morozov S.V., Jiang D., Zhang Y., Dubonos S.V., Grigorieva I.V., Firsov A.A. (2004). Electric Field Effect in Atomically Thin Carbon Films. Science.

[15] Li L., Yu Y., Ye G.J., Ge Q., Ou X., Wu H., Feng D., Chen X.H., Zhang Y. (2014). Black Phosphorus Field-Effect Transistors. Nat. Nanotechnol..

[16] Bridgman P.W. (1914). Two New Modifications of Phosphorus. J. Am. Chem. Soc..

[17] Sun X., Liu X., Yin J., Yu J., Li Y., Hang Y., Zhou X., Yu M., Li J., Tai G. (2017). Two-Dimensional Boron Crystals: Structural Stability, Tunable Properties, Fabrications and Applications. Appl. Phys. Lett..

[18] Niu T., Zhou W., Zhou D., Hu X., Zhang S., Zhang K., Zhou M., Fuchs H., Zeng H. (2019). Atomic Structure of Antimonene through Interface Design. Adv. Mater..

[19] Ares P., Palacios J.J., Abellan G., Gomez-Herrero J., Zamora F. (2018). Recent Progress on Antimonene: A New Bidimensional Material. Adv. Mater..

[20] Li L.H., Chen Y. (2016). Atomically Thin Boron Nitride: Unique Properties and Applications. Adv. Funct. Mater..

[21] Han W.-Q., Wu L., Zhu Y., Watanabe K., Taniguchi T. (2008). Structure of Chemically Derived Mono- and Few-Atomic-Layer Boron Nitride Sheets. Appl. Phys. Lett..

[22] Kalantar-zadeh K., Ou J.Z., Daeneke T., Mitchell A., Sasaki T., Fuhrer M.S. (2016). Two-Dimensional and Layered Transition Metal Oxides. Appl. Mater. Today.

[23] Chung C., Kim Y.K., Shin D., Ryoo S.R., Hong B.H., Min D.H. (2013). Biomedical Applications of Graphene and Graphene Oxide. Acc. Chem. Res..

[24] Malisz K., Świeczko-Żurek B. (2023). Graphene Production and Biomedical Applications: A Review. Crystals.

[25] Cheng C., Li D. (2013). Solvated Graphenes: An Emerging Class of Functional Soft Materials. Adv. Mater..

[26] Liu Y., Dong X., Chen P. (2012). Biological and Chemical Sensors Based on Graphene Materials. Chem. Soc. Rev..

[27] Chen Y., Tan C., Zhang H., Wang L. (2015). Two-Dimensional Graphene Analogues for Biomedical Applications. Chem. Soc. Rev..

[28] Kurapati R., Kostarelos K., Prato M., Bianco A. (2016). Biomedical Uses for 2D Materials Beyond Graphene: Current Advances and Challenges Ahead. Adv. Mater..

[29] Gan X., Zhao H., Quan X. (2017). Two-Dimensional MoS_2_: A Promising Building Block for Biosensors. Biosens. Bioelectron..

[30] Barua S., Dutta H.S., Gogoi S., Devi R., Khan R. (2018). Nanostructured MoS_2_-Based Advanced Biosensors: A Review. ACS Appl. Nano Mater..

[31] Dalila R.N., Md Arshad M.K., Gopinath S.C.B., Norhaimi W.M.W., Fathil M.F.M. (2019). Current and Future Envision on Developing Biosensors Aided by 2D Molybdenum Disulfide (MoS_2_) Productions. Biosens. Bioelectron..

[32] Hu Y., Huang Y., Tan C., Zhang X., Lu Q., Sindoro M., Huang X., Huang W., Wang L., Zhang H. (2017). Two-Dimensional Transition Metal Dichalcogenide Nanomaterials for Biosensing Applications. Mater. Chem. Front..

[33] Yadav V., Roy S., Singh P., Khan Z., Jaiswal A. (2019). 2D MoS_2_-Based Nanomaterials for Therapeutic, Bioimaging, and Biosensing Applications. Small.

[34] Yang B., Chen Y., Shi J. (2018). Material Chemistry of Two-Dimensional Inorganic Nanosheets in Cancer Theranostics. Chem.

[35] Gong L., Yan L., Zhou R., Xie J., Wu W., Gu Z. (2017). Two-Dimensional Transition Metal Dichalcogenide Nanomaterials for Combination Cancer Therapy. J. Mater. Chem. B.

[36] Wang X., Chang J., Wu C., Yang L., Bhaduri S.B., Webster T.J. (2019). 7-MoS_2_-Based Biomaterials for Cancer Therapy. Biomaterials in Translational Medicine.

[37] Liang W., Luo X. (2020). Theoretical Studies of MoS_2_ and Phosphorene Drug Delivery for Antituberculosis Drugs. J. Phys. Chem. C.

[38] Li B.L., Setyawati M.I., Chen L., Xie J., Ariga K., Lim C.-T., Garaj S., Leong D.T. (2017). Directing Assembly and Disassembly of 2D MoS_2_ Nanosheets with DNA for Drug Delivery. ACS Appl. Mater. Interfaces.

[39] Cheng L., Wang X., Gong F., Liu T., Liu Z. (2020). 2D Nanomaterials for Cancer Theranostic Applications. Adv. Mater..

[40] Wang J., Yang M., Cui W., Zhao X. (2019). 11-Two-Dimensional Nanomaterials in Cancer Theranostics. Theranostic Bionanomaterials.

[41] Teo W.Z., Chng E.L., Sofer Z., Pumera M. (2014). Cytotoxicity of Exfoliated Transition-Metal Dichalcogenides (MoS_2_, WS_2_, and WSe_2_) is Lower Than That of Graphene and its Analogues. Chem. Eur. J..

[42] Qin S., Li K., Zhu J., Xu H., Ali N., Rahimi-Iman A., Wu H. (2021). A New Strategy to Improve the Performance of MoS_2_-Based 2D Photodetector by Synergism of Colloidal CuInS_2_ Quantum Dots and Surface Plasma Resonance of Noble Metal Nanoparticles. J. Alloy. Compd..

[43] Shi Y., Zhang Q., Zhai T.-T., Zhou Y., Yang D.-R., Wang F.-B., Xia X.-H. (2018). Localized Surface Plasmon Resonance Enhanced Label-Free Photoelectrochemical Immunoassay by Au-MoS_2_ Nanohybrid. Electrochim. Acta.

[44] El Barghouti M., Akjouj A., Mir A. (2020). MoS_2_–Graphene Hybrid Nanostructures Enhanced Localized Surface Plasmon Resonance Biosensors. Opt. Laser Technol..

[45] Younis M.R., Wang C., An R., Wang S., Younis M.A., Li Z.-Q., Wang Y., Ihsan A., Ye D., Xia X.-H. (2019). Low Power Single Laser Activated Synergistic Cancer Phototherapy Using Photosensitizer Functionalized Dual Plasmonic Photothermal Nanoagents. ACS Nano.

[46] Toh R.J., Sofer Z., Luxa J., Sedmidubsky D., Pumera M. (2017). 3R Phase of MoS_2_ and WS_2_ Outperforms the Corresponding 2H Phase for Hydrogen Evolution. Chem. Commun..

[47] Jiao Y., Hafez A.M., Cao D., Mukhopadhyay A., Ma Y., Zhu H. (2018). Metallic MoS_2_ for High Performance Energy Storage and Energy Conversion. Small.

[48] Manzeli S., Dumcenco D., Migliato Marega G., Kis A. (2019). Self-Sensing, Tunable Monolayer MoS_2_ Nanoelectromechanical Resonators. Nat. Commun..

[49] Li X., Zhu H. (2015). Two-Dimensional MoS_2_: Properties, Preparation, and Applications. J. Mater..

[50] Dai Z., Jin W., Grady M., Sadowski J.T., Dadap J.I., Osgood R.M., Pohl K. (2017). Surface Structure of Bulk 2H-MoS_2_ (0001) and Exfoliated Suspended Monolayer MoS_2_: A Selected Area Low Energy Electron Diffraction Study. Surf. Sci..

[51] Siao M.D., Shen W.C., Chen R.S., Chang Z.W., Shih M.C., Chiu Y.P., Cheng C.M. (2018). Two-Dimensional Electronic Transport and Surface Electron Accumulation in MoS_2_. Nat. Commun..

[52] Seivane L.F., Barron H., Botti S., Marques M.A., Rubio A., Lopez-Lozano X. (2013). Atomic and Electronic Properties of Quasi-One-Dimensional MoS_2_ Nanowires. J. Mater. Res..

[53] Elizondo-Villarreal N., Velázquez-Castillo R., Galván D.H., Camacho A., José Yacamán M. (2007). Structure and Catalytic Properties of Molybdenum Sulfide Nanoplatelets. Appl. Catal. A Gen..

[54] Tahersima M.H., Birowosuto M.D., Ma Z., Coley W.C., Valentin M.D., Naghibi Alvillar S., Lu I.H., Zhou Y., Sarpkaya I., Martinez A. (2017). Testbeds for Transition Metal Dichalcogenide Photonics: Efficacy of Light Emission Enhancement in Monomer vs Dimer Nanoscale Antennae. ACS Photonics.

[55] Saleem U., Permatasari F.A., Iskandar F., Ogi T., Okuyama K., Darma Y., Zhao M., Loh K.P., Rusydi A., Coquet P. (2017). Surface Plasmon Enhanced Nitrogen-Doped Graphene Quantum Dot Emission by Single Bismuth Telluride Nanoplates. Adv. Opt. Mater..

[56] Hou S., Tobing L.Y.M., Wang X., Xie Z., Yu J., Zhou J., Zhang D., Dang C., Coquet P., Tay B.K. (2019). Manipulating Coherent Light–Matter Interaction: Continuous Transition between Strong Coupling and Weak Coupling in MoS_2_ Monolayer Coupled with Plasmonic Nanocavities. Adv. Opt. Mater..

[57] Johari P., Shenoy V.B. (2012). Tuning the Electronic Properties of Semiconducting Transition Metal Dichalcogenides by Applying Mechanical Strains. ACS Nano.

[58] Kuc A., Zibouche N., Heine T. (2011). Influence of Quantum Confinement on the Electronic Structure of the Transition Metal Sulfide TS_2_. Phys. Rev. B.

[59] Venkata Subbaiah Y.P., Saji K.J., Tiwari A. (2016). Atomically Thin MoS_2_: A Versatile Nongraphene 2D Material. Adv. Funct. Mater..

[60] Halim S.N.M., Zuikafly S.N.F., Taib M.F.M., Ahmad F. First Principles Study on Electronic and Optical Properties of Graphene/MoS_2_ for Optoelectronic Application. Proceedings of the 2020 IEEE International Conference on Semiconductor Electronics (ICSE).

[61] Nalwa H.S. (2020). A Review of Molybdenum Disulfide (MoS_2_) Based Photodetectors: From Ultra-Broadband, Self-Powered to Flexible Devices. RSC Adv..

[62] Eda G., Yamaguchi H., Voiry D., Fujita T., Chen M., Chhowalla M. (2011). Photoluminescence from Chemically Exfoliated MoS_2_. Nano Lett..

[63] Mak K.F., Lee C., Hone J., Shan J., Heinz T.F. (2010). Atomically Thin MoS_2_: A New Direct-Gap Semiconductor. Phys. Rev. Lett..

[64] Sundaram R.S., Engel M., Lombardo A., Krupke R., Ferrari A.C., Avouris P., Steiner M. (2013). Electroluminescence in Single Layer MoS_2_. Nano Lett..

[65] Splendiani A., Sun L., Zhang Y., Li T., Kim J., Chim C.Y., Galli G., Wang F. (2010). Emerging Photoluminescence in Monolayer MoS_2_. Nano Lett..

[66] Yin Z., Li H., Li H., Jiang L., Shi Y., Sun Y., Lu G., Zhang Q., Chen X., Zhang H. (2012). Single-Layer MoS_2_ Phototransistors. ACS Nano.

[67] Li G., Zhang D., Qiao Q., Yu Y., Peterson D., Zafar A., Kumar R., Curtarolo S., Hunte F., Shannon S. (2016). All The Catalytic Active Sites of MoS_2_ for Hydrogen Evolution. J. Am. Chem. Soc..

[68] Makarova M., Okawa Y., Aono M. (2012). Selective Adsorption of Thiol Molecules at Sulfur Vacancies on MoS_2_(0001), Followed by Vacancy Repair via S–C Dissociation. J. Phys. Chem. C.

[69] Li H., Wang S., Sawada H., Han G.G., Samuels T., Allen C.S., Kirkland A.I., Grossman J.C., Warner J.H. (2017). Atomic Structure and Dynamics of Single Platinum Atom Interactions with Monolayer MoS_2_. ACS Nano.

[70] Vijayan A., Sandhyarani N. (2022). Enhancing the Catalytic Activity of Bulk MoS_2_ Towards Hydrogen Evolution Reaction by the Formation of MoS_2_-MoO_3_-Re_2_O_7_ Heterostructure. J. Colloid Interface Sci..

[71] Xu Y., Ge R., Yang J., Li J., Li S., Li Y., Zhang J., Feng J., Liu B., Li W. (2022). Molybdenum Disulfide (MoS_2_)-Based Electrocatalysts for Hydrogen Evolution Reaction: From Mechanism to Manipulation. J. Energy Chem..

[72] Stergiou A., Tagmatarchis N. (2018). Molecular Functionalization of Two-Dimensional MoS_2_ Nanosheets. Chem. Eur. J..

[73] Ataca C., Ciraci S. (2011). Functionalization of Single-Layer MoS_2_ Honeycomb Structures. J. Phys. Chem. C.

[74] Kalantar-zadeh K., Ou J.Z. (2015). Biosensors Based on Two-Dimensional MoS_2_. ACS Sens..

[75] Yu X., Prévot M.S., Sivula K. (2014). Multiflake Thin Film Electronic Devices of Solution Processed 2D MoS_2_ Enabled by Sonopolymer Assisted Exfoliation and Surface Modification. Chem. Mat..

[76] Chhowalla M., Shin H.S., Eda G., Li L.J., Loh K.P., Zhang H. (2013). The Chemistry of Two-Dimensional Layered Transition Metal Dichalcogenide Nanosheets. Nat. Chem..

[77] Eda G., Fujita T., Yamaguchi H., Voiry D., Chen M., Chhowalla M. (2012). Coherent Atomic and Electronic Heterostructures of Single-Layer MoS_2_. ACS Nano.

[78] Tan C., Zhang H. (2015). Two-Dimensional Transition Metal Dichalcogenide Nanosheet-Based Composites. Chem. Soc. Rev..

[79] Voiry D., Goswami A., Kappera R., Castro e Silva C.d.C., Kaplan D., Fujita T., Chen M., Asefa T., Chhowalla M. (2015). Covalent Functionalization of Monolayered Transition Metal Dichalcogenides by Phase Engineering. Nat. Chem..

[80] Gómez-Muñoz I., Laghouati S., Torres-Cavanillas R., Morant-Giner M., Vassilyeva N.V., Forment-Aliaga A., Giménez-Marqués M. (2021). Fast Polymeric Functionalization Approach for the Covalent Coating of MoS_2_ Layers. ACS Appl. Mater. Interfaces.

[81] Gan F., Dong N., Liu Z., Jia H., Wang J., Chen Y. (2019). Organic Small Molecule Covalently Functionalized Molybdenum Disulfide Hybrid Material for Optical Limiting. Bull. Chem. Soc. Jpn..

[82] Liu T., Wang C., Gu X., Gong H., Cheng L., Shi X., Feng L., Sun B., Liu Z. (2014). Drug Delivery with Pegylated MoS_2_ Nano-Sheets for Combined Photothermal and Chemotherapy of Cancer. Adv. Mater..

[83] Kwon H.J., Shin K., Soh M., Chang H., Kim J., Lee J., Ko G., Kim B.H., Kim D., Hyeon T. (2018). Large-Scale Synthesis and Medical Applications of Uniform-Sized Metal Oxide Nanoparticles. Adv. Mater..

[84] Huang A., He Y., Zhou Y., Zhou Y., Yang Y., Zhang J., Luo L., Mao Q., Hou D., Yang J. (2019). A Review of Recent Applications of Porous Metals and Metal Oxide in Energy Storage, Sensing and Catalysis. J. Mater. Sci..

[85] Falcaro P., Ricco R., Yazdi A., Imaz I., Furukawa S., Maspoch D., Ameloot R., Evans J.D., Doonan C.J. (2016). Application of Metal and Metal Oxide Nanoparticles@MOFs. Coord. Chem. Rev..

[86] Chavali M.S., Nikolova M.P. (2019). Metal Oxide Nanoparticles and Their Applications in Nanotechnology. Discov. Appl. Sci..

[87] Mphuthi N., Sikhwivhilu L., Ray S.S. (2022). Functionalization of 2D MoS_2_ Nanosheets with Various Metal and Metal Oxide Nanostructures: Their Properties and Application in Electrochemical Sensors. Biosensors.

[88] Lim W.Q., Gao Z. (2016). Plasmonic Nanoparticles in Biomedicine. Nano Today.

[89] Lee S., Sun Y., Cao Y., Kang S.H. (2019). Plasmonic Nanostructure-Based Bioimaging and Detection Techniques at the Single-Cell Level. TrAC Trends Anal. Chem..

[90] Acunzo A., Scardapane E., De Luca M., Marra D., Velotta R., Minopoli A. (2022). Plasmonic Nanomaterials for Colorimetric Biosensing: A Review. Chemosensors.

[91] Duan H., Wang T., Su Z., Pang H., Chen C. (2022). Recent Progress and Challenges in Plasmonic Nanomaterials. Nanotechnol. Rev..

[92] Borghei Y.S., Hosseinkhani S., Ganjali M.R. (2022). “Plasmonic Nanomaterials”: An Emerging Avenue in Biomedical and Biomedical Engineering Opportunities. J. Adv. Res..

[93] Çimen D., Ünal S., Denizli A. (2025). Nanoparticle-Assisted Plasmonic Sensors: Recent Developments in Clinical Applications. Anal. Biochem..

[94] Zhou J., Yang T., Chen J., Wang C., Zhang H., Shao Y. (2020). Two-Dimensional Nanomaterial-Based Plasmonic Sensing Applications: Advances and Challenges. Coord. Chem. Rev..

[95] Mak K.F., Sfeir M.Y., Wu Y., Lui C.H., Misewich J.A., Heinz T.F. (2008). Measurement of the Optical Conductivity of Graphene. Phys. Rev. Lett..

[96] Nair R.R., Blake P., Grigorenko A.N., Novoselov K.S., Booth T.J., Stauber T., Peres N.M., Geim A.K. (2008). Fine Structure Constant Defines Visual Transparency of Graphene. Science.

[97] Huang S., Song C., Zhang G., Yan H. (2016). Graphene Plasmonics: Physics and Potential Applications. Nanophotonics.

[98] Zhang N., Han C., Fu X., Xu Y.-J. (2018). Function-Oriented Engineering of Metal-Based Nanohybrids for Photoredox Catalysis: Exerting Plasmonic Effect and Beyond. Chem.

[99] Erwin W.R., Zarick H.F., Talbert E.M., Bardhan R. (2016). Light Trapping in Mesoporous Solar Cells with Plasmonic Nanostructures. Energy Environ. Sci..

[100] Yesilkoy F., Terborg R.A., Pello J., Belushkin A.A., Jahani Y., Pruneri V., Altug H. (2018). Phase-Sensitive Plasmonic Biosensor Using a Portable and Large Field-of-View Interferometric Microarray Imager. Light Sci. Appl..

[101] Verma S., Pathak A.K., Rahman B.M.A. (2024). Review of Biosensors Based on Plasmonic-Enhanced Processes in the Metallic and Meta-Material-Supported Nanostructures. Micromachines.

[102] Jeong H.-H., Choi E., Ellis E., Lee T.-C. (2019). Recent Advances in Gold Nanoparticles for Biomedical Applications: From Hybrid Structures to Multi-Functionality. J. Mater. Chem. B.

[103] Zheng W., Chiamori H.C., Liu G.L., Lin L., Chen F.F. (2012). Nanofabricated Plasmonic Nano-Bio Hybrid Structures in Biomedical Detection. Nanotechnol. Rev..

[104] Yao C., Zhang L., Wang J., He Y., Xin J., Wang S., Xu H., Zhang Z. (2016). Gold Nanoparticle Mediated Phototherapy for Cancer. J. Nanomater..

[105] Mukha I., Chepurna O., Vityuk N., Khodko A., Storozhuk L., Dzhagan V., Zahn D.R.T., Ntziachristos V., Chmyrov A., Ohulchanskyy T.Y. (2021). Multifunctional Magneto-Plasmonic Fe_3_O_4_/Au Nanocomposites: Approaching Magnetophoretically-Enhanced Photothermal Therapy. Nanomaterials.

[106] Fan X., Zheng W., Singh D.J. (2014). Light Scattering and Surface Plasmons on Small Spherical Particles. Light Sci. Appl..

[107] Battie Y., Resano-Garcia A., Chaoui N., En Naciri A. (2015). Optical Properties of Plasmonic Nanoparticles Distributed in Size Determined from a Modified Maxwell-Garnett-Mie Theory. Phys. Status Solidi C.

[108] Borah R., Verbruggen S.W. (2020). Silver–Gold Bimetallic Alloy versus Core–Shell Nanoparticles: Implications for Plasmonic Enhancement and Photothermal Applications. J. Phys. Chem. C.

[109] Sadeghi F., Soleimanian V., Ghasemi M., Ahangar H.A. (2023). Simulation of Fe_2_O_3_/Au@MoS_2_ Plasmonic Core/Shell@Shell Nanocomposites: Optical Properties and Photothermal Application. Mater. Today Commun..

[110] Yang W., Li J., Huang Y. (2020). Time-Domain Finite Element Method and Analysis for Modeling of Surface Plasmon Polaritons. Comput. Methods Appl. Mech. Eng..

[111] Yang Z., Li Q., Ruan F., Li Z., Ren B., Xu H., Tian Z. (2010). FDTD for Plasmonics: Applications in Enhanced Raman Spectroscopy. Chin. Sci. Bull..

[112] Ke Q., Chen L., Fang B., Chen Y., Zhang W. (2021). Simulating Electric Field Intensity Distribution of Lspr Based on Gold Nanobipyramids. Mater. Today Commun..

[113] Jain P.K., Lee K.S., El-Sayed I.H., El-Sayed M.A. (2006). Calculated Absorption and Scattering Properties of Gold Nanoparticles of Different Size, Shape, and Composition:  Applications in Biological Imaging and Biomedicine. J. Phys. Chem. B.

[114] Battie Y., Resano-Garcia A., Chaoui N., Zhang Y., En Naciri A. (2014). Extended Maxwell-Garnett-Mie Formulation Applied to Size Dispersion of Metallic Nanoparticles Embedded in Host Liquid Matrix. J. Chem. Phys.

[115] Bhardwaj S., Barr J., Chaffin E., Huang X., Wang Y. (2019). Near-Field and Far-Field Optical Properties of Magnetic Plasmonic Core-Shell Nanoparticles with Non-Spherical Shapes: A Discrete Dipole Approximation Study. AIP Adv..

[116] Zhang H., Shen Y., Xu Y., Zhu H., Lei M., Zhang X., Xu M. (2013). Effective Medium Theory for Two-Dimensional Random Media Composed of Core–Shell Cylinders. Opt. Commun..

[117] Reshetnyak V.Y., Pinkevych I.P., Sluckin T.J., Urbas A.M., Evans D.R. (2018). Effective Medium Theory for Anisotropic Media with Plasmonic Core-Shell Nanoparticle Inclusions. Eur. Phys. J. Plus.

[118] Xu Y., Zhang Y., Li C., Ye Z., Bell S.E.J. (2023). SERS as a Probe of Surface Chemistry Enabled by Surface-Accessible Plasmonic Nanomaterials. Acc. Chem. Res..

[119] Campion A., Kambhampati P. (1998). Surface-Enhanced Raman Scattering. Chem. Soc. Rev..

[120] Kleinman S.L., Frontiera R.R., Henry A.-I., Dieringer J.A., Van Duyne R.P. (2013). Creating, Characterizing, and Controlling Chemistry with SERS Hot Spots. Phys. Chem. Chem. Phys..

[121] Potara M., Gabudean A.-M., Astilean S. (2011). Solution-Phase, Dual Lspr-Sers Plasmonic Sensors of High Sensitivity and Stability Based on Chitosan-Coated Anisotropic Silver Nanoparticles. J. Mater. Chem..

[122] Vlasov A.V., Maliar N.L., Bazhenov S.V., Nikelshparg E.I., Brazhe N.A., Vlasova A.D., Osipov S.D., Sudarev V.V., Ryzhykau Y.L., Bogorodskiy A.O. (2020). Raman Scattering: From Structural Biology to Medical Applications. Crystals.

[123] Lussier F., Brulé T., Vishwakarma M., Das T., Spatz J.P., Masson J.-F. (2016). Dynamic-SERS Optophysiology: A Nanosensor for Monitoring Cell Secretion Events. Nano Lett..

[124] Yang Y., Pan R., Tian S., Gu C., Li J. (2020). Plasmonic Hybrids of MoS_2_ and 10-nm Nanogap Arrays for Photoluminescence Enhancement. Micromachines.

[125] Samy O., Zeng S., Birowosuto M.D., El Moutaouakil A. (2021). A Review on MoS_2_ Properties, Synthesis, Sensing Applications and Challenges. Crystals.

[126] Zhou X., Hao H., Zhang Y.-J., Zheng Q., Tan S., Zhao J., Chen H.-B., Chen J.-J., Gu Y., Yu H.-Q. (2021). Patterning of Transition Metal Dichalcogenides Catalyzed by Surface Plasmons with Atomic Precision. Chem.

[127] Nam K., Im J., Han G.H., Park J.Y., Kim H., Park S., Yoo S., Haddadnezhad M., Ahn J.S., Park K.-D. (2024). Photoluminescence of MoS_2_ on Plasmonic Gold Nanoparticles Depending on the Aggregate Size. ACS Omega.

[128] Zhang N., Zheng Y.J., Zhu L.R., Zou H.L., Luo H.Q., Li N.B., Li B.L. (2023). Molybdenum Disulfide Nanostructures Coupled with Metal Plasmonics for Improved Electronic and Photonic Performances. J. Mater. Chem. C.

[129] Huang X., Zeng Z., Bao S., Wang M., Qi X., Fan Z., Zhang H. (2013). Solution-Phase Epitaxial Growth of Noble Metal Nanostructures on Dispersible Single-Layer Molybdenum Disulfide Nanosheets. Nat. Commun..

[130] Shi Y., Huang J.K., Jin L., Hsu Y.T., Yu S.F., Li L.J., Yang H.Y. (2013). Selective Decoration of Au Nanoparticles on Monolayer MoS_2_ Single Crystals. Sci. Rep..

[131] Shi Y., Huang W.M., Li J., Zhou Y., Li Z.Q., Yin Y.C., Xia X.H. (2020). Site-Specific Electrodeposition Enables Self-Terminating Growth of Atomically Dispersed Metal Catalysts. Nat. Commun..

[132] Yang P., Wang D., Zhao X., Quan W., Jiang Q., Li X., Tang B., Hu J., Zhu L., Pan S. (2022). Epitaxial Growth of Inch-Scale Single-Crystal Transition Metal Dichalcogenides Through the Patching of Unidirectionally Orientated Ribbons. Nat. Commun..

[133] Li B.L., Zou H.L., Luo H.Q., Leong D.T., Li N.B. (2020). Layered MoS_2_ Defect-Driven in Situ Synthesis of Plasmonic Gold Nanocrystals Visualizes the Planar Size and Interfacial Diversity. Nanoscale.

[134] Su S., Xu Y., Sun Q., Gu X., Weng L., Wang L. (2018). Noble Metal Nanostructure-Decorated Molybdenum Disulfide Nanocomposites: Synthesis and Applications. J. Mater. Chem. B.

[135] Li B.L., Zou H.L., Tian J.K., Chen G., Wang X.H., Duan H., Li X.L., Shi Y., Chen J.R., Li L.J. (2019). Principle of Proximity: Plasmonic Hot Electrons Motivate Donator-Adjacent Semiconductor Defects with Enhanced Electrocatalytic Hydrogen Evolution. Nano Energy.

[136] Yuan Y., Yang B., Jia F., Song S. (2019). Reduction Mechanism of Au Metal Ions into Au Nanoparticles on Molybdenum Disulfide. Nanoscale.

[137] Seravalli L., Bosi M., Fiorenza P., Panasci S.E., Orsi D., Rotunno E., Cristofolini L., Rossi F., Giannazzo F., Fabbri F. (2021). Gold Nanoparticle Assisted Synthesis of MoS_2_ Monolayers by Chemical Vapor Deposition. Nanoscale Adv..

[138] Li Y., DiStefano J.G., Murthy A.A., Cain J.D., Hanson E.D., Li Q., Castro F.C., Chen X., Dravid V.P. (2017). Superior Plasmonic Photodetectors Based on Au@MoS_2_ Core-Shell Heterostructures. ACS Nano.

[139] Li Y., Hao S., DiStefano J.G., Murthy A.A., Hanson E.D., Xu Y., Wolverton C., Chen X., Dravid V.P. (2018). Site-Specific Positioning and Patterning of MoS_2_ Monolayers: The Role of Au Seeding. ACS Nano.

[140] Shi D., Jia G., Yao J. (2020). Formation of an Ag/MoS_2_ Composite Structure through Photothermal Conversion. AIP Adv..

[141] Garoli D., Mosconi D., Miele E., Maccaferri N., Ardini M., Giovannini G., Dipalo M., Agnoli S., De Angelis F. (2018). Hybrid Plasmonic Nanostructures Based on Controlled Integration of MoS_2_ Flakes on Metallic Nanoholes. Nanoscale.

[142] Zuo P., Jiang L., Li X., Li B., Ran P., Li X., Qu L., Lu Y. (2018). Metal (Ag, Pt)–MoS_2_ Hybrids Greenly Prepared Through Photochemical Reduction of Femtosecond Laser Pulses for SERS and HER. ACS Sustain. Chem. Eng..

[143] Yaiwong P., Jakmunee J., Pimalai D., Ounnunkad K., Bamrungsap S. (2024). An Electrochemical/SERS Dual-Mode Immunosensor Using TMB/Au Nanotag and Au@2D-MoS_2_ Modified Screen-Printed Electrode for Sensitive Detection of Prostate Cancer Biomarker. Colloid Surf. B Biointerfaces.

[144] Liu Y., Nie Y., Wang M., Zhang Q., Ma Q. (2020). Distance-Dependent Plasmon-Enhanced Electrochemiluminescence Biosensor Based on MoS_2_ Nanosheets. Biosens. Bioelectron..

[145] Nor S.N.S., Rasanang N.S., Karman S.B., Zaman W.S.W.K., Harun S.W., Arof H. (2024). Incorporation of Thiol-Modified and Carboxyl-MoS_2_ Films as Support Layer in Surface Plasmon Resonance Sensing Platform for SARS-CoV-2 Spike Protein Detection. IEEE Sens. J..

[146] Maji S.K., Yu S., Chung K., Sekkarapatti Ramasamy M., Lim J.W., Wang J., Lee H., Kim D.H. (2018). Synergistic Nanozymetic Activity of Hybrid Gold Bipyramid–Molybdenum Disulfide Core@Shell Nanostructures for Two-Photon Imaging and Anticancer Therapy. ACS Appl. Mater. Interfaces.

[147] Xu Y., Kang Q., Yang B., Chen B., He M., Hu B. (2020). A Nanoprobe Based on Molybdenum Disulfide Nanosheets and Silver Nanoclusters for Imaging and Quantification of Intracellular Adenosine Triphosphate. Anal. Chim. Acta.

[148] Liu C., Li Y., Li W., Fan Y., Zhou W., Xiao C., Yu P., Liu Y., Liu X., Huang Z. (2024). LSPR- Enhanced Photoresponsive Antibacterial Efficiency of Bi/MoS_2_-Loaded Fibrin Gel for Management of Diabetic Wounds. Int. J. Biol. Macromol..

[149] Wang W., Wang J., Li J., Cao S., Shi J. (2023). In Situ Growth of MoS_2_@Aunrs Nanoparticles with Synergistically Enhanced NIR Response for Controlled Drug Release. Mater. Today Commun..

[150] Lv J., Li B., Luo T., Nie C., Pan W., Ge X., Zheng J., Rui Y., Zheng L. (2023). Selective Photothermal Therapy Based on Lipopolysaccharide Aptamer Functionalized MoS_2_ Nanosheet-Coated Gold Nanorods for Multidrug-Resistant Pseudomonas aeruginosa Infection. Adv. Healthc. Mater..

[151] Yougbare S., Mutalik C., Chung P.F., Krisnawati D.I., Rinawati F., Irawan H., Kristanto H., Kuo T.R. (2021). Gold Nanorod-Decorated Metallic MoS_2_ Nanosheets for Synergistic Photothermal and Photodynamic Antibacterial Therapy. Nanomaterials.

[152] Liu L., Wang J., Tan X., Pang X., You Q., Sun Q., Tan F., Li N. (2017). Photosensitizer Loaded PEG-MoS_2_–Au Hybrids for CT/NIRF Imaging-Guided Stepwise Photothermal and Photodynamic Therapy. J. Mater. Chem. B.

[153] Wei F., Cui X., Wang Z., Dong C., Li J., Han X. (2021). Recoverable Peroxidase-Like Fe_3_O_4_@MoS_2_-Ag Nanozyme with Enhanced Antibacterial Ability. Chem. Eng. J..

[154] Rodriguez-da-Silva S., El-Hachimi A.G., Lopez-de-Luzuriaga J.M., Rodriguez-Castillo M., Monge M. (2023). Boosting the Catalytic Performance of AuAg Alloyed Nanoparticles Grafted on MoS_2_ Nanoflowers through NIR-Induced Light-to-Thermal Energy Conversion. Nanomaterials.

[155] Bassi A., Schmid B., Huisken J. (2015). Optical Tomography Complements Light Sheet Microscopy for in TOTO Imaging of Zebrafish Development. Development.

[156] Mayer J., Robert-Moreno A., Danuser R., Stein J.V., Sharpe J., Swoger J. (2014). OPTiSPIM: Integrating Optical Projection Tomography in Light Sheet Microscopy Extends Specimen Characterization to Nonfluorescent Contrasts. Opt. Lett..

[157] Chen H., Rogalski M.M., Anker J.N. (2012). Advances in Functional X-Ray Imaging Techniques and Contrast Agents. Phys. Chem. Chem. Phys..

[158] Pushie M.J., Pickering I.J., Korbas M., Hackett M.J., George G.N. (2014). Elemental and Chemically Specific X-ray Fluorescence Imaging of Biological Systems. Chem. Rev..

[159] Keevend K., Coenen T., Herrmann I.K. (2020). Correlative Cathodoluminescence Electron Microscopy Bioimaging: Towards Single Protein Labelling with Ultrastructural Context. Nanoscale.

[160] Sun Y., Yu M., Liang S., Zhang Y., Li C., Mou T., Yang W., Zhang X., Li B., Huang C. (2011). Fluorine-18 Labeled Rare-Earth Nanoparticles for Positron Emission Tomography (PET) Imaging of Sentinel Lymph Node. Biomaterials.

[161] DeMarco V.P., Ordonez A.A., Klunk M., Prideaux B., Wang H., Zhuo Z., Tonge P.J., Dannals R.F., Holt D.P., Lee C.K. (2015). Determination of [11c]Rifampin Pharmacokinetics within Mycobacterium Tuberculosis-Infected Mice by Using Dynamic Positron Emission Tomography Bioimaging. Antimicrob. Agents Chemother..

[162] Kondo Y., Nonaka H., Takakusagi Y., Sando S. (2021). Design of Nuclear Magnetic Resonance Molecular Probes for Hyperpolarized Bioimaging. Angew. Chem. Int. Ed. Engl..

[163] Zhou T., Jia L., Luo Y.-F., Xu J., Chen R.-H., Ge Z.-J., Ma T.-L., Chen H., Zhu T.-F. (2016). Multifunctional Nanocomposite Based on Halloysite Nanotubes for Efficient Luminescent Bioimaging and Magnetic Resonance Imaging. Int. J. Nanomed..

[164] Celli J.P., Spring B.Q., Rizvi I., Evans C.L., Samkoe K.S., Verma S., Pogue B.W., Hasan T. (2010). Imaging and Photodynamic Therapy: Mechanisms, Monitoring, and Optimization. Chem. Rev..

[165] Melancon M.P., Zhou M., Li C. (2011). Cancer Theranostics with Near-Infrared Light-Activatable Multimodal Nanoparticles. Acc. Chem. Res..

[166] Yang K., Xu H., Cheng L., Sun C., Wang J., Liu Z. (2012). In Vitro and in Vivo Near-Infrared Photothermal Therapy of Cancer Using Polypyrrole Organic Nanoparticles. Adv. Mater..

[167] Cui X., Ruan Q., Zhuo X., Xia X., Hu J., Fu R., Li Y., Wang J., Xu H. (2023). Photothermal Nanomaterials: A Powerful Light-to-Heat Converter. Chem. Rev..

[168] Verma S.S., Bhatia P., Chakraborti S. (2021). Plasmonic Photothermal Therapy (PPTT) of Cancer. Handbook of Oxidative Stress in Cancer: Therapeutic Aspects.

[169] Sethulekshmi A.S., Saritha A., Joseph K., Aprem A.S., Sisupal S.B. (2022). MoS_2_ Based Nanomaterials: Advanced Antibacterial Agents for Future. J. Control. Release.

[170] Tu C.-Y., Wu J.M. (2021). Localized Surface Plasmon Resonance Coupling with Piezophototronic Effect for Enhancing Hydrogen Evolution Reaction with Au@MoS_2_ Nanoflowers. Nano Energy.

[171] Dolmans D.E.J.G.J., Fukumura D., Jain R.K. (2003). Photodynamic Therapy for Cancer. Nat. Rev. Cancer.

[172] Kamata H., Honda S.-I., Maeda S., Chang L., Hirata H., Karin M. (2005). Reactive Oxygen Species Promote TNFα-Induced Death and Sustained JNK Activation by Inhibiting MAP Kinase Phosphatases. Cell.

[173] Simon H.U., Haj-Yehia A., Levi-Schaffer F. (2000). Role of Reactive Oxygen Species (ROS) in Apoptosis Induction. Apoptosis.

[174] Liu M., Zhu H., Wang Y., Sevencan C., Li B.L. (2021). Functionalized MoS_2_-Based Nanomaterials for Cancer Phototherapy and Other Biomedical Applications. ACS Mater. Lett..

